# Ruminosignatures associated with methane emissions and feed efficiency across geographies and cattle breeds

**DOI:** 10.1093/ismejo/wrag185

**Published:** 2026-07-12

**Authors:** Ioanna-Theoni Vourlaki, Ori Furman, Ilma Tapio, Le Luo Guan, Sinéad M Waters, David Kenny, Paul Smith, Stuart F Kirwan, David Kelly, Ross Evans, Raquel Quintanilla, Miriam Piles, Antonio Reverter, Pâmela A Alexandre, Fuyong Li, Philip C Garnsworthy, Paolo Bani, Phillip B Pope, Diego P Morgavi, Itzhak Mizrahi, Yuliaxis Ramayo-Caldas

**Affiliations:** Animal Breeding and Genetics Program, IRTA, Torre Marimon, 08140 Caldes de Montbui, Spain; Department of Life Sciences, Ben-Gurion University of the Negev, Beer-Sheva 84105, Israel; Genomics and Breeding, Natural Resources Finland (Luke), Jokioinen 31600, Finland; Faculty of Land and Food Systems, The University of British Columbia, V6T 1Z4 Vancouver, Canada; School of Biological and Chemical Sciences and Ryan Institute, University of Galway, Galway H91 TK33, Ireland; Animal and Bioscience Department, Teagasc Grange, Dunsany, Co. Meath C15 PW93, Ireland; Animal and Bioscience Department, Teagasc Grange, Dunsany, Co. Meath C15 PW93, Ireland; Department of Animal Science, University of California, Davis, CA 95616, United States; Irish Cattle Breeding Federation, Link Road, Ballincolling, Co. Cork, P31 D452, Ireland; Irish Cattle Breeding Federation, Link Road, Ballincolling, Co. Cork, P31 D452, Ireland; Animal Breeding and Genetics Program, IRTA, Torre Marimon, 08140 Caldes de Montbui, Spain; Animal Breeding and Genetics Program, IRTA, Torre Marimon, 08140 Caldes de Montbui, Spain; CSIRO Agriculture and Food, St. Lucia, Brisbane, QLD 4067, Australia; CSIRO Agriculture and Food, St. Lucia, Brisbane, QLD 4067, Australia; Department of Animal Science and Technology, College of Animal Sciences, Zhejiang University, Hangzhou 310058, PR China; School of Biosciences, University of Nottingham, Sutton Bonington Campus, Loughborough LE12 5RD, United Kingdom; Department of Animal Science, Food and Nutrition, Università Cattolica del Sacro Cuore, 29122 Piacenza, Italy; Centre for Microbiome Research, School of Biomedical Sciences, Queensland University of Technology (QUT), Translational Research Institute, Woolloongabba, QLD 4102, Australia; Faculty of Chemistry, Biotechnology and Food Science, Norwegian University of Life Sciences, 1432 Ås, Norway; Université Clermont Auvergne, INRAE, VetAgro Sup, UMR Herbivores, Saint-Genès-Champanelle F-63122, France; Department of Life Sciences, Ben-Gurion University of the Negev, Beer-Sheva 84105, Israel; Animal Breeding and Genetics Program, IRTA, Torre Marimon, 08140 Caldes de Montbui, Spain

**Keywords:** agriculture, breeding, cattle, feed efficiency, heritability, methane, rumen microbiota, Ruminosignatures

## Abstract

The cattle rumen microbiota represents a complex and dynamic ecosystem whose organization and relationship to host phenotypes are important for food security and environmental sustainability. We analyzed rumen microbiota profiles from 2496 cattle representing five breeds and production systems across five countries, identifying microbial co-abundance groups termed Ruminosignatures. We detected 14 distinct Ruminosignatures, including 2 observed across all populations dominated by Prevotella and UBA2810. Additional Ruminosignatures showed breed- and diet-specific patterns and collectively explained 96%–99% of variance in rumen microbial composition. Integrative cross-country analysis confirmed 10 out of 14 Ruminosignatures identified in cohort-specific analyses. Several Ruminosignatures were associated with methane emissions and feed efficiency traits and were partially under host genetic control, with heritability estimates ranging from 0.09 to 0.58. Structural equation modeling revealed consistent negative genetic and phenotypic correlations between the UBA2810-dominated Ruminosignature (RS_UBA2) and methane emissions across cohorts (rg = −0.40 to −0.65), with structural coefficients concordant in sign across all populations, supporting the expected direction of phenotypic response to selection on RS_UBA2. Meta-analysis confirmed positive associations of RS_UBA2 with average daily gain and negative associations with methane-related traits and feed conversion ratio. Functional genome-based predictions suggested RS_UBA2 may reduce methanogenesis through alternative hydrogen utilization pathways competing with methanogenic archaea. Production system type influenced both Ruminosignature occurrence and relationships with host phenotypes, emphasizing the relevance of context-specific strategies for microbiome modulation. Our findings highlight the potential of the Ruminosignatures framework for microbiome-informed breeding programs aimed at improving feed efficiency while reducing the environmental impact of cattle production.

## Introduction

Cattle production plays a central role in global food systems, with beef and dairy sectors contributing significantly to food security and economic sustainability [1]. Similar to other livestock species, cattle production faces global challenges related to the need to improve profitability and decrease its environmental impact. In this scenario, methane (CH₄) emissions and feed efficiency are critical traits that significantly impact both the productivity and environmental sustainability of cattle production [2–4]. Reductions in CH₄ emissions and improvements in feed efficiency can be achieved through a combination of management, nutritional strategies, and selective breeding [5, 6]. Although management and nutritional strategies can have immediate effects, breeding provides a more permanent solution through genetic improvement. Although feed efficiency has been successfully incorporated into breeding programs and methane emissions are beginning to be included [7, 8], the phenotypic measurement of these traits at individual level remains challenging and costly for large-scale implementation. Central to both traits is the rumen microbiome, which mediates feed conversion and simultaneously produces CH_4_, via specific microbial taxa such as methanogenic archaea, protozoa, and associated bacteria. Integrating microbial signals that are linked to ruminal CH_4_ production into selection models offer an opportunity to enhance the accuracy of breeding programs [5, 9]. Most rumen microbiome studies have focused on specific cattle populations, such as European Aberdeen Angus × Limousin steers [10], Nordic Red dairy cows [11], or selected European and North American herds reviewed recently [5, 12]. This narrow scope limits the generalizability of findings across breeds and production systems, underscoring the need for multi-country datasets to assess microbiome potential and develop context-specific, microbiome-informed strategies. Microbiome compositions are complex, sparse, and exhibit multivariate dependencies, making the analysis of these datasets particularly challenging. Instead of treating hundreds or thousands of individual microbial taxa as separate signals, the approach proposed by Frioux *et al*. [13] aims to reduce complexity and capture ecosystem-level dynamics by identifying co-abundant and co-occurring microbial guilds. Microbial guilds are defined as groups of microorganisms that display coordinated abundance patterns, often reflecting shared ecological roles and niche preferences, and consistently co-abundant behavior [14]. This approach has been demonstrated in humans [13] and pigs [15] and employs non-negative matrix factorization (NMF) to reduce dataset dimensionality by identifying latent co-abundance patterns that capture shared variance across taxa. Although traditional dimensionality reduction methods, such as principal component analysis (PCA) or ruminotypes, have been widely employed in studies of ruminant microbiota [16–18], they present certain limitations. PCA, although widely used, forces its components to be orthogonal and assigns both positive and negative loadings to taxa. This can mask biologically meaningful patterns and may combine taxa in ways that are not easy to interpret ecologically. Microbial guilds, however, often exhibit overlapping or correlated activity patterns driven by ecological interactions. On the other hand, Ruminotype-based approaches classify samples into groups based on their dominant taxa, assigning each sample to one group only. Although these methods reduce dimensionality and can stratify samples to identify the taxon driving each cluster, they do not consider the ecological context. Consequently, the information they provide is limited in comparison. This study identifies microbial co-abundance and co-occurring groups defined as Ruminosignatures (RS) and demonstrates how these groups, comprising rumen microbes that share consistent abundance patterns across individuals in diverse geographies and cattle populations, can be used as variables associated with host traits such as methane emissions and feed efficiency.

## Material and methods

### Study populations and experimental design

The current study incorporates data from 2496 animals from independent populations in five different countries that represent different cattle breeds and production systems. The discovery dataset consisted 818 cows: 409 Holstein lactating cows from the UK and 409 from Italy [19]. A cohort consisting of 120 Holstein cows from France set [18] was used as independent validation. Two additional cohorts of beef and crossbreed cattle were employed as independent validation sets. The first one consisted of 709 multi-breeds (203 Angus, 114 Charolais, and 392 Kinsella composite hybrids) which were raised under standardized feedlot conditions at the University of Alberta’s Roy Berg Kinsella Research Ranch, Canada [20]. The second one includes 849 crossbreds from Ireland [21]. All animals were fed standardized diets appropriate for their production stage, with beef cattle receiving growth-phase-specific rations, crossbred from Ireland fed with a high-energy concentrate-based diet [21, 22], and dairy cows maintained on total mixed rations for at least 14 days prior to sampling. Animal handling complied with the ethical standards and regulations established by Canada and the European Union directives on animal experimentation (European Commission, 2010). Ethical approval for the different studies can be found in the references cited above.

### Sample collection and processing

Rumen contents were collected via esophagus in all cohorts using distinct but complementary protocols. For beef cattle, approximately 50 ml of rumen fluid was obtained via oro-gastric tubing before morning feeding at 293 ± 0.6 days of age, immediately frozen on dry ice, and stored at −80°C [20]. Irish cattle were sampled using the FLORA rumen scoop sampling device (Guelph, ON, Canada). Dairy cow rumen samples were collected using a Ruminator device (Profs Products, Wittybreut, Germany), with the first liter discarded to avoid saliva contamination, followed by collection of 500 ml of representative rumen fluid between 2 and 5 h post-feeding [18, 19]. Samples were processed for immediate pH measurement, with aliquots preserved for volatile fatty acid analysis following standard procedures.

### Phenotypic measurements

**Figure 1:**
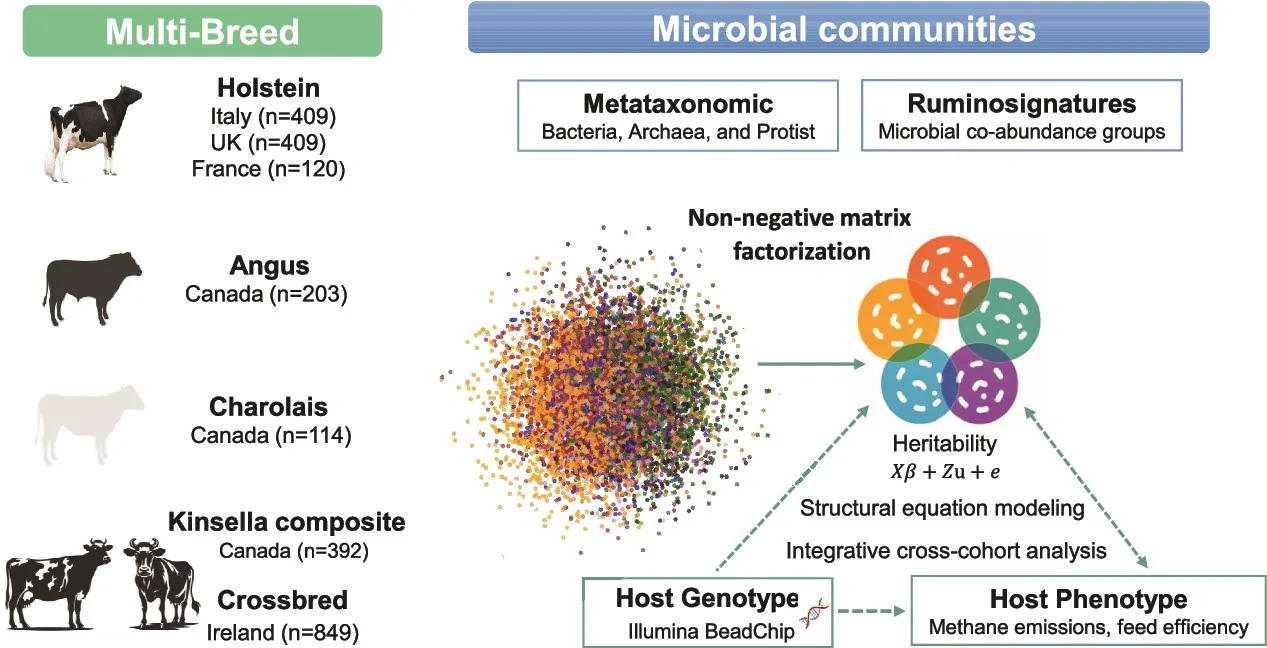
Summary of cattle breeds populations and analytical approach used in the study.

Comprehensive production data were collected across the datasets (Fig. 1). The beef and crossbred cattle trials recorded detailed feed efficiency metrics including dry matter intake (DMI), average daily gain (ADG), residual feed intake (RFI), and feed conversion ratio (FCR); CH_4_ emissions using GreenFeed emission monitoring system (C-Lock Ltd., Ireland) only; and acetate and propionate levels were monitored for the Canadian beef cattle. The dairy cow trials monitored milk production parameters, body weight, and CH_4_ emissions using either breath sampling during milking (UK) or GreenFeed (France, Italy). Table 1 presents a full description of the traits included in the association analysis, whereas a summary of the datasets used across the analyses can be found on Supplementary Table 1.

**Table 1:** Summary statistics of the analyzed traits related to methane production, growth, and feed efficiency across countries and genetic backgrounds.

Trait	Country (breed)	No. of samples	Mean (SD)
Methane production (CH₄ g/d)	UK (Holstein)	407	430 (74)
	Italy (Holstein)	403	385 (106)
	France (Holstein)	65	506 (58)
	Ireland (Crossbred)	875	233 (51)
Methane production expressed as grams per kilogram of dry matter intake (CH₄ g/kg DMI)	UK (Holstein)	402	19 (5)
	Italy (Holstein)	403	18 (5)
	Ireland (Crossbred)	883	20 (4.7)
Energy-corrected milk yield relative to dry matter intake (ECM kg/DMI)	UK (Holstein)	402	1.5 (0.24)
	Italy (Holstein)	397	1.4 (0.33)
Average daily gain (ADG)	Ireland (Crossbred)	779	1.5 (0.4)
	Canada (Angus)	183	1.3 (0.44)
	Canada (Charolais)	91	1.4 (0.33)
	Canada (Kinsella composite)	297	1.2 (0.35)
Feed conversion ratio (FCR)	Ireland (Crossbred)	779	7.9 (2.3)
	Canada (Angus)	183	7.8 (2.5)
	Canada (Charolais)	91	7.8 (1.8)
	Canada (Kinsella composite)	297	7.6 (1.9)
Residual feed intake (RFI)	Canada (Angus)	183	0.0 (0.6)
	Canada (Charolais)	91	0.0 (0.6)
	Canada (Kinsella composite)	297	0.0 (0.53)
Residual feed intake (calculated from NRC equations; RFI.NRC)	UK (Holstein)	402	−2.4 (3.24)
	Italy (Holstein)	397	−0.5 (3.9)
Acetate-to-propionate ratio (Ace:Prop)	Canada (Angus)	200	3.5 (1)
	Canada (Charolais)	113	2 (0.7)
	Canada (Kinsella composite)	392	2.8 (0.9)

### Microbial DNA extraction and sequencing

Total genomic DNA was extracted from rumen samples using optimized protocols for each study. The beef cattle samples in Canada underwent automated extraction using the QIAGEN BioSprint 96 workstation. Irish samples were extracted using the DNeasy PowerSoil Pro Kit (QIAGEN GmbH) whereas dairy cattle samples employed a bead-beating protocol combined with the QIAamp DNA Stool Mini Kit (Qiagen). For bacterial and archaeal community profiling, the V1–V3 (UK, IT, Canada for bacteria), V3–V5 (France), V4 (Ireland), and V6–V8 (Canada for archaeal methanogens) regions of the 16S rRNA genes were amplified using established primer sets for bacteria and archaea. As described by Wallace *et al*. [19], the discovery dairy cohort additionally targeted protozoal 18S rRNA markers. All amplicons were generated on a MiSeq System (Illumina).

### Bioinformatic processing

Sequence data were processed through a standardized QIIME2 pipeline [23]. Raw reads underwent quality filtering (Phred score ≥ 20), adapter removal, and chimera detection. Amplicon sequence variants were generated at 99% similarity, with subsequent removal of low-abundance variants (<0.0001% total counts) and samples containing <8000 reads. Taxonomic classification was performed using a customized GreenGenes2 (release 2024.09, [24]) reference database trained for the specific primer sets employed within each dataset, whereas the SILVA 138.2 database [25] was used specifically for taxonomic assignation of protozoan sequences. Report of sequencing-depth statistics for each cohort can be found on Supplementary Table 2.

### Identification of Ruminosignatures

Following the approach described by Frioux *et al*. [13], the existence of RS was assessed using NMF with a normalized genera relative abundance table as input. The table was normalized using total sum scaling, such that each column represented an individual sample; values were divided by the column sum so that each column summed to 1. NMF is an unsupervised dimensionality reduction technique that factorizes a non-negative input matrix into the product of two lower-rank matrices: ***W***, defined as the weight of the genera in each RS, and ***H***, which describe the RS abundance across samples. NMF identifies latent variables in the data capturing co-abundant patterns while performing a parts-based representation. Due to differences in primer sets across datasets, NMF was applied independently to each cohort. The optimal number of RS was accessed via a nine-fold bi-cross validation [26] as suggested in Frioux *et al*. [13] for selecting the best rank in outer product models. For each of the nine folds, the model was trained on 8/9 of the data and evaluated on the remaining 1/9. This cross-validation procedure was repeated 200 times for a range of latent variables (*k*) from 2 to 30, with the input matrix randomly shuffled at each iteration. The most suitable number of RS was selected by monitoring the evaluation criteria associated with each *k*, including explained variance, and cosine similarity. To perform bi-cross validation, we used the Python script “***bicv_rank.py***” from the repository of Frioux *et al*. [13]. The L1 regularization parameter (alpha) was estimated using the companion script “***bicv_regu.py***,” with the final value chosen according to the rank considered optimal. The corresponding alpha value for each dataset is provided in Supplementary Table 3. The analysis was carried out with the Python implementation proposed by Frioux *et al*. [13] in the same repository. The computations were run through the terminal in Python 3.9 [27], making use of SciPy v1.7.3 [28] and Scikit-learn v0.24.1 [29]. The analysis was performed on normalized genus-level relative abundance tables. Normalization was carried out by dividing the counts in each column (representing an individual sample) by the total sum of counts in that column, so that each column summed to 1 and all values were positive. Each configuration was executed for 100 iterations, with a maximum of 2000 updates, random initialization, a defined regularization ratio, the multiplicative update algorithm, and the Kullback–Leibler divergence as the β-loss function. Regularization was applied simultaneously to both the ***W*** and ***H*** matrices. We investigated the correlation patterns between RS and the abundance of methanogenic archaea. Particularly, for the Holstein discovery dataset, links with the abundance of protozoal ruminal communities was also explored.

## Results

### Ruminosignatures profiles across Holstein populations

NMF analysis revealed the existence of six RS in the Holstein discovery population. The optimal number of RS (*k* = 6) was determined based on the plateau observed in both cosine similarity and explained variance metrics (Supplementary Fig. 4), beyond which additional factors yielded negligible improvement. This model explained 99% of the variance and achieved a cosine similarity of 99% from the original genus abundance table (Supplementary Table 4).

**Figure 2:**
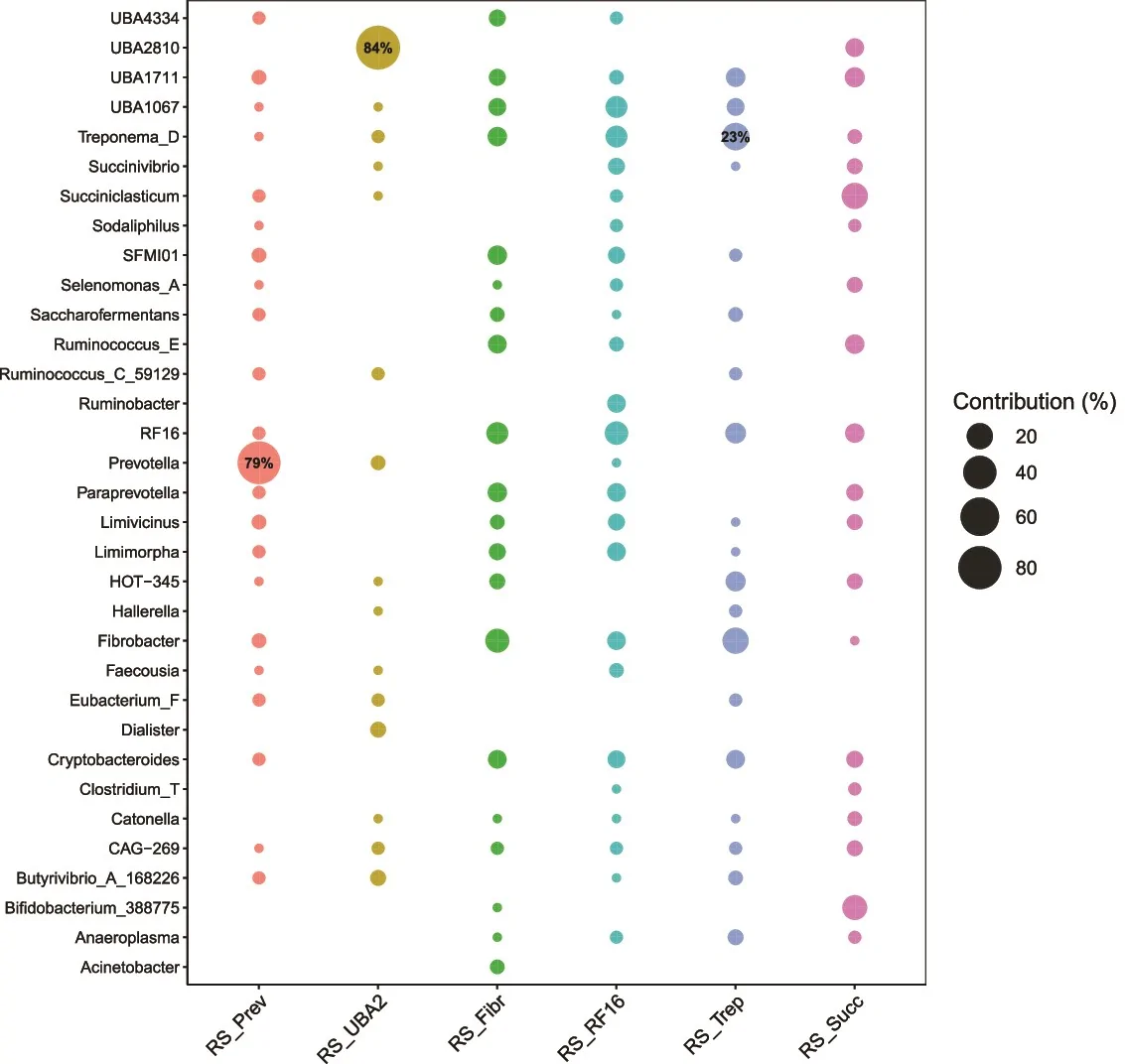
Relative genera composition of Ruminosignatures in the Holstein discovery set. Only genera with a contribution >1% in at least one of the Ruminosignatures are shown.

The driving genera were defined as those with the highest contributions within each microbial co-abundance group. Following this criterion, the taxonomic composition of the six RSs was driven by *Prevotella* (RS_Prev), *UBA2810* (RS_UBA2), *Fibrobacter* (RS_Fibr), *RF16* (RS_RF16), *Treponema* (RS_Trep), and *Succiniclasticum* (RS_Succ). The composition of the six RSs and their corresponding genus contributions are shown, displaying only genera with a relative contribution >1% in at least one RS (Fig. 2). The RS profiles vary in both taxonomic structure and contribution. Although RS_Prev and RS_UBA2 are largely dominated by a few genera, the others (RS_Fibr, RS_RF16, RS_Trep, and RS_Succ) represent microbial groups composed of multiple genera with low to moderate contributions. To validate the initial findings, we applied the same NMF-based approach to an independent Holstein dataset from France. In this cohort, the optimal rank was determined to be *k* = 7 (Supplementary Fig. 5), capturing 99% of the variance in bacterial genus abundances and achieving a cosine similarity of 99% (Supplementary Table 4). Four RS including RS_Prev, RS_UBA2, RS_Fibr, and RS_RF16 were consistent with those identified in the discovery dataset. The remaining three were specific to the French population and dominated by *Sodaphilus* (RS_Soda), *Succinivibrio* (RS_Succ), and *Butyrivibrio_A_168226* (RS_Buty) genera. The genus-level composition of each RS is shown in Supplementary Fig. 6, with the dominant genera contributing between 14% and 74% of the total abundance within their respective microbial groups. In agreement with the results from the discovery dataset, RS_Prev and RS_UBA2 were dominated by a few genera, whereas the others represented microbial groups composed of multiple genera with low to moderate contributions (Supplementary Fig. 6).

### Ruminosignatures profiles across Canadian and Irish populations

Results from the Canadian cohort suggested the existence of eight RS (Supplementary Fig. 7), collectively explaining 96% of the total variance in genus-level abundance data and achieving a cosine similarity of 98% (Supplementary Table 4). The main RS driving variation in this dataset were RS_Prev, RS_UBA2, RS_Rumi, RS_Succ, RS_SFMI, RS_Limi, RS_Soda, and RS_Cryp, corresponding to the dominant genera *Prevotella, UBA2810, Ruminococcus, Succiniclasticum, SFMI, Limimorpha, Sodaliphilus*, and *Cryptobacteroides*, respectively (Supplementary Fig. 8). As shown in Supplementary Fig. 8, and consistent with observations from Holstein populations, RS_Prev and RS_UBA2 were again primarily driven by their dominant genera, contributing 86% and 93% of the respective RS composition. Within the Irish crossbreed population, seven RS (Supplementary Fig. 9) were identified accounting for 97% of the total variance in the original genera matrix and capturing 98% of the cosine similarity (Supplementary Table 4). The taxonomic composition of the main RS was dominated by RS_Prev, RS_UBA2, RS_Rumi, RS_Succ, RS_SFMI, RS_RF16, and *Sharpea* (RS_Shar). As observed in other populations, RS_Prev and RS_UBA2 were primarily driven by their respective dominant genus, whereas the remaining RS consisted of microbial groups composed of genera with low to moderate contributions (Supplementary Fig. 10).

### Population-level variation in Ruminosignature profiles

**Figure 3:**
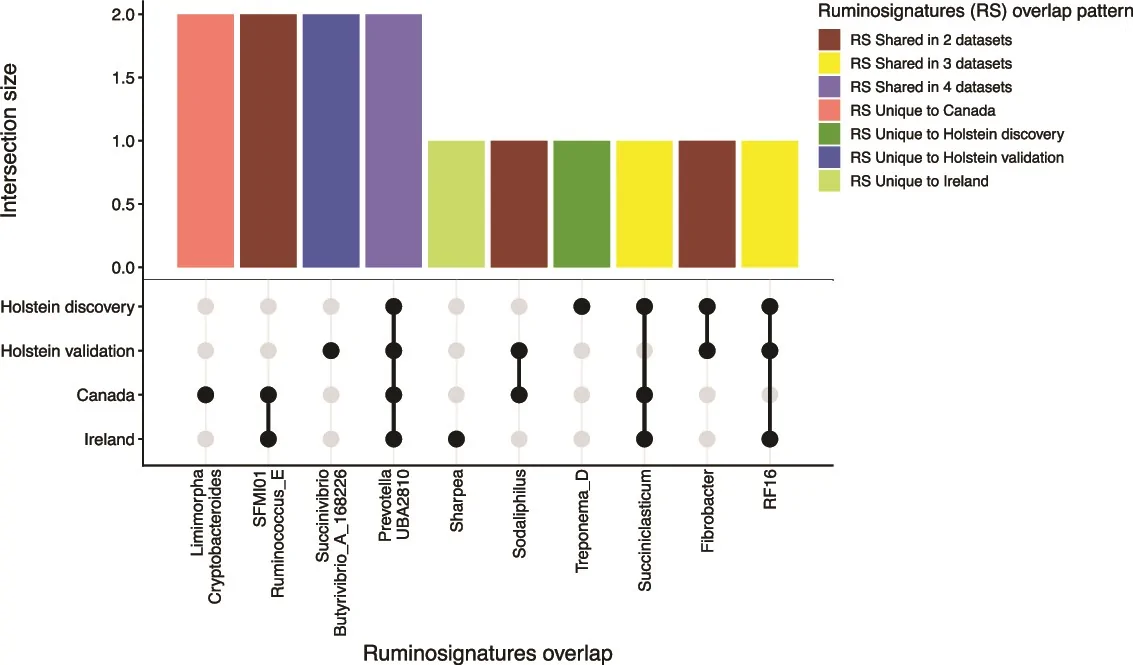
Comparison of Ruminosignatures intersection across all the datasets. The bar plot (top) shows the number of Ruminosignatures that are unique to each dataset or shared across 2, 3, or all 4 datasets. The UpSet plot (bottom) displays the overlap structure of individual Ruminosignatures across the Canada, Ireland, Holstein discovery, and Holstein validation datasets. Filled circles indicate the presence of a given Ruminosignature in each dataset. Together, the figure summarizes both dataset-specific and common Ruminosignatures across the study cohorts.

The comparison across all datasets revealed two RSs that were common across geographies, breed and populations: RS_Prev and RS_UBA2 (Fig. 3). Another two (driven by *RF16* and *Succiniclasticum*) were common across three of the four datasets, whereas RS_Fibr, RS_SFMI, RS_Rumi, and RS_Soda were detected in at least two populations. As observed in Fig. 3, some RSs were specific to populations, indicating that diet, breed, or management practices may contribute to their population-specific distribution. Particularly, RS_Trep was exclusive to the Holstein discovery dataset, whereas the RS driven by *Sharpea* was observed only in the Irish crossbreed population. RSs driven by *Limimorpha* and *Cryptobacteroides* were unique to the Canadian cohort, and *Succinivibrio* and *Butyrivibrio* were exclusively found in the French Holstein population (Supplementary Fig. 6). When comparing Canadian beef cattle with the Irish cohort and Holstein discovery dataset, three common RSs were identified, dominated by *Prevotella, UBA2810*, and *Succiniclasticum*. In contrast, comparison with the French Holstein population revealed a different overlap, for the shared RS dominated by *Sodaliphilus*. In addition, an integrative cross-cohort analysis including all available datasets was performed to evaluate whether the microbial signatures identified in the cohort-specific analyses were globally consistent or cohort dependent (see Supplementary Information Methods for details). This analysis identified 15 RS, confirming that, after correction for confounding factors (fixed effects, sequencing depth), 10 of the 14 initially reported RS (Fig. 3) remained major contributors to the assembly of the ruminal microbial ecosystem. Across cohorts, microbial structure was primarily driven by *Prevotella* and *UBA2810* (Supplementary Fig. 11). In addition, RSs driven by genera previously identified in the initial per-dataset analyses (Fig. 3) were also recovered, including *RF16, SM1F1, Ruminococcus_E, Cryptobacteroides, Treponema, Butyrivibrio_A_168226, Limimorpha*, and *Succiniclasticum*. The remaining RS (RS_Shar, RS_Fibr, RS_Soda, and the RS driven by *Succinivibrio*) were not retained as major drivers across cohorts, suggesting that these may represent cohort-specific microbial signatures, consistent with the observations from the original per-dataset analyses. In addition, *Limivicinus, Paraprevotella, UBA1711*, and *CAG-269*, which were previously identified as dominant contributors in the independent cohort analyses, were detected in the cross-cohort analysis as driven RS (Supplementary Fig. 12). Overall, these results support the robustness of the NMF framework and confirm that most RS identified in the cohort-specific analyses are reproducible under an integrative cross-cohort framework.

### Pairwise cross-cohort similarity analysis

**Figure 4:**
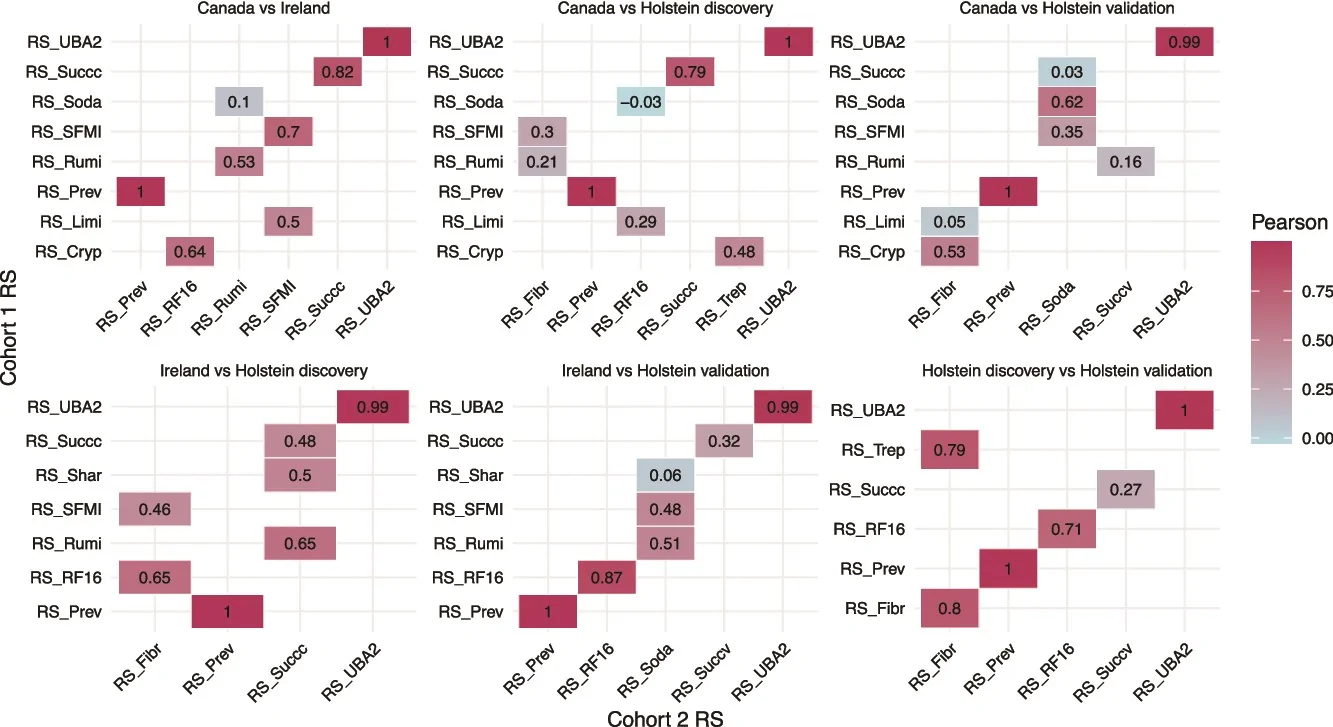
Cross-cohort Ruminosignature similarity. Heatmaps visualize pairwise Pearson correlation coefficients between Ruminosignatures identified independently in each cohort. Rows correspond to Ruminosignatures from cohort 1 and columns correspond to Ruminosignatures from cohort 2.

To assess the reproducibility and transferability of RS across different cattle populations, we compared the ***W*** matrices obtained from NMF for each cohort (see Supplementary Information Methods). Results quantitatively revealed that several signatures are highly conserved across cattle populations. RS dominated by the same genera showed almost perfect similarity, with Pearson correlations close to 1. For example, core RS dominated by the *Prevotella genus* in one cohort was nearly identical to the corresponding RS in the other cohort, and RS_UBA2 displayed similarly high correspondence. Beyond the core, the average pattern reveals strong and positive correlations within each comparison when comparing shared Ruminosignatures (Canada vs Ireland *r*_mean_ = 0.81; Canada vs Holstein_discovery *r*_mean_ = 0.93; Canada vs Holstein_validation *r*_mean_ = 0.87; Ireland vs Holstein_discovery *r*_mean_ = 0.82; Ireland vs Holstein_validation *r*_mean_ = 0.95; Holstein_discovery vs Holstein_validation *r*_mean_ = 0.88). These results confirm the qualitative observation indicating that some Ruminosignatures represent robust and reproducible microbial assemblages across different management systems (Fig. 4).

### Association with methane emissions and feed efficiency

**Figure 5:**
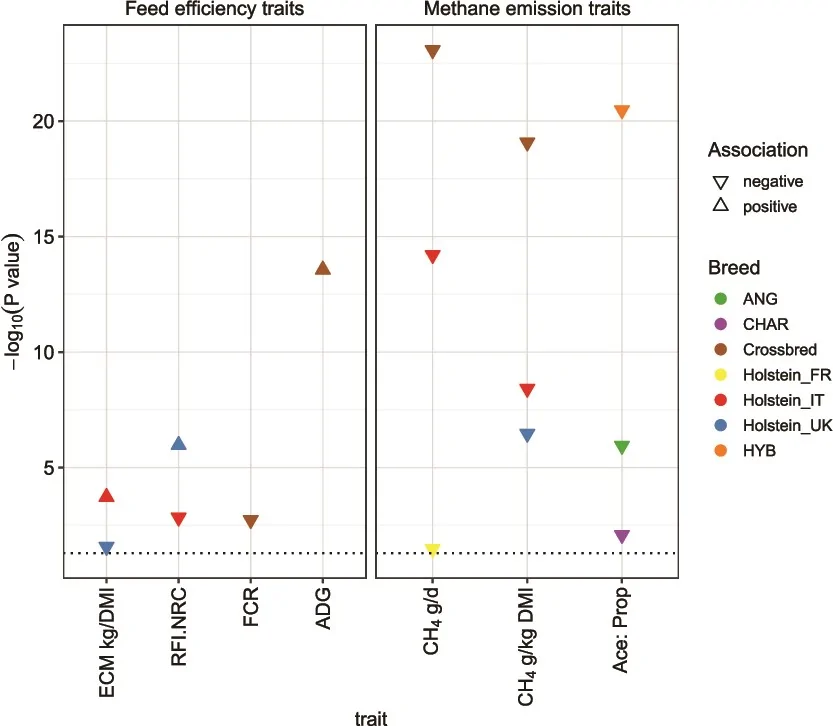
Linear associations between RS_UBA2 abundances and methane or host performance traits. Arrows indicate the direction of the relationship between the Ruminosignature driven by UBA2 and host trait. Only significant associations are shown, although the traits were measured in additional breeds not displayed in the plot.

Beyond characterizing the number and taxonomic composition of the RS, we investigated their associations with CH₄ emissions and feed efficiency traits. To this end, linear models correcting for confounders (see Supplementary Information Methods for details) were conducted to explore the relationship between the RS abundance and CH₄ and feed efficiency–related parameters. We observed that RS_UBA2 was consistently negatively associated (*P* < .05) with CH₄ emission across all breeds (France, Ireland, Italy, and the UK), and with Ace:Prop in Canadian beef populations (Fig. 5). RS_Prev showed a negative association with CH_4_ in Holstein animals but not in the Irish crossbred population (Supplementary Fig. 13). In contrast, RS_RF16 generally exhibited positive associations with CH₄ emissions, except in the Canadian population, where the Ace:Prop was employed as proxy of CH_4_ emission (Supplementary Fig. 13). In the same dataset from Canada, RS_SFMI and RS_Rumi were significantly (*P* < .05) positively associated with a lower Ace:Prop. Although the Canadian animals were fed rumensin and the model accounts for dietary differences, the specific effects of rumensin on the ruminal microbiota remain unclear. Further research is needed to determine whether it alters community composition or the abundance of microorganisms. We also observed inverse correlation patterns indicating opposing abundance relationships among RS associated with CH₄. For example, in the Holstein populations, RS_UBA2 showed a negative correlation with the abundance of RS_RF16 (*r* = −0.65, *P* < .001). RS_UBA2 was also negatively correlated (*r* = −0.39, *P* < .001) with the abundance of the methanogenic archaea *Methanobrevibacter*, whereas the abundance of RS_RF16 was positively correlated with *Methanobrevibacter* (*r* = 0.37, *P* < .001). In the Irish cohort, RS_UBA2 negatively correlated with the abundance of RS_Rumi (*r* = −0.42, *P* < .001) and negatively with *Methanobrevibacter* (*r* = −0.25, *P* < .01), whereas the abundance of RS_Rumi positively correlated with *Methanobrevibacter* (*r* = 0.49, *P* < .001). The same pattern, (RS_UBA2 vs RS_Rumi *r* = −0.24, *P* < .01; RS_Rumi vs *Methanobrevibacter r* = 0.32; *P* < .001) was confirmed in the Canadian dataset.

Regarding feed efficiency traits, RS_UBA2 was negatively associated with FCR and positively associated with ADG in the crossbred Irish population (Fig. 5). This indicates that crossbreed animals from Ireland with higher abundance of RS_UBA2 tend to utilize feed more efficiently resulting in higher daily growth rates whereas emitting less CH₄. A similar association pattern was observed in Holstein cattle from Italy, where RS_UBA2 showed a positive association with energy-corrected milk per dry matter intake (ECM kg/DMI) and a negative association with RFI. In contrast, Holstein cows from UK exhibited the opposite trend, suggesting that the relationship between RS_UBA2 and feed efficiency in Holstein cows may be influenced by population-specific factors such as diet, environment, or management practices. To summarize the association of RS_UBA2 with methane-related traits across multiple countries, we performed a meta-analysis (for method details see Supplementary information). The meta-analysis identified robust, cross-country associations of RS_UBA2 with several traits. Specifically, ADG, CH₄ g/d, CH₄ g/kg DMI, and the Ace:Prop ratio showed strong, consistent effects (all *P*_combined <10^−14^) in the expected directions: positive for ADG, negative for methane-related traits, and negative for the Ace:Prop ratio. These associations remained significant after both FDR and Bonferroni correction, underscoring their robustness and reproducibility across countries. In contrast, ECM kg/DMI and RFI_NRC were no longer significant in the meta-analysis (*P*_combined >.2) suggesting that the decrease in CH₄ was not consistently associated with changes in productivity or energy efficiency. FCR showed a moderate negative association (*P*_combined ≈ .002) that remained significant after multiple-testing correction, suggesting a potentially consistent, though less robust, effect across populations. Overall, the weighted, sign-aware two-tailed Stouffer meta-analysis based on two-sided *P*-values method provides a statistically rigorous framework for summarizing RS_UBA2 associations, effectively highlighting reproducible cross-country signals while minimizing the risk of false positives attributable to heterogeneous or weak effects (Supplementary Table 5).

### Host genetic control on Ruminosignatures abundance

We estimated the posterior mean heritability (${\hat{\mathrm{h}}}_{\mathrm{a}}^2$) of the abundance of each RS across the beef (Canada), beef-dairy crossbred (Ireland), and Holstein (UK and Italy) datasets (Table 2, see Supplementary Information Methods for details and Supplementary Figures 1-3). France validation dataset was not included in this analysis because of the small number of available animals. When comparing heritability estimates between common and dataset-specific RSs, the highest values were observed in the Irish crossbred dataset, for which the abundance of RSs driven by *UBA2810, Ruminococcus_E, Succiniclasticum*, and *SFMI* showed posterior mean heritability of 0.48, 0.49, 0.51, and 0.58, respectively. Additionally, the RS driven by *Sharpea* exhibited a moderate heritability estimate of 0.38 in this crossbred population. The Holstein discovery dataset showed intermediate heritability values for the different RS abundances (${\hat{h}}_{\mathrm{a}}^2$ = 0.15–0.27), whereas the Canadian dataset displayed the lowest estimates (${\hat{h}}_{\mathrm{a}}^2$ = 0.09–0.17).

**Table 2:** Summary of posterior mean of heritability and 95% highest posterior density interval (HPD95) for each Ruminosignature across datasets.

RS	Country	${\hat{h}}_a^2$	HPD95 [lower upper]
RS_Prev	Canada	0.11	[0.04 0.19]
	Holstein	0.17	[0.1 0.25]
	Ireland	0.17	[0.05 0.30]
RS_UBA2	Canada	0.14	[0.05 0.24]
	Holstein	0.27	[0.15 0.39]
	Ireland	0.48	[0.33 0.63]
RS_SFMI	Canada	0.17	[0.07 0.29]
	Ireland	0.58	[0.44 0.71]
RS_Rumi	Canada	0.13	[0.06 0.22]
	Ireland	0.49	[0.34 0.65]
RS_Succ	Canada	0.15	[0.08 0.25]
	Holstein	0.15	[0.1 0.22]
	Ireland	0.51	[0.36 0.65]
RS_Limi	Canada	0.13	[0.06 0.21]
RS_Soda	Canada	0.12	[0.05 0.21]
RS_Cryp	Canada	0.09	[0.04 0.16]
RS_Fibr	Holstein	0.25	[0.15 0.35]
RS_RF16	Holstein	0.24	[0.13 0.36]
	Ireland	0.24	[0.11 0.39]
RS_Trep	Holstein	0.27	[0.15 0.38]
RS_Shar	Ireland	0.34	[0.16 0.52]

### Directional relationships between RS_UBA2 and methane traits

The bivariate recursive structural equation model (SEM; for a detailed description see Supplementary Information) revealed predominantly negative genetic correlations between RS_UBA2 abundance and methane-related traits across populations, with estimates ranging from −0.40 to −0.65 (Supplementary Table 6). Phenotypic correlations were also negative across all populations (Holstein: −0.34; Ireland: −0.52; Canada: −0.47) consistent in sign with the corresponding genetic correlations, supporting the biological plausibility of the estimated genetic relationships. The recursive structural coefficient, representing the directional effect of RS_UBA2 abundance on methane-related traits, showed variable levels of statistical support across populations. Strong evidence for a non-zero recursive effect was detected in the Canadian cohort (*N* = 709, *Z* = −12.66), while Holstein approached the threshold (*N* = 818, *Z* = −1.99). In Ireland (*N* = 849, *Z* = −0.92), the structural coefficient did not reach statistical significance. The sign of the structural coefficient was concordant with the sign of the genetic correlation across all three populations (all negative), indicating that higher RS_UBA2 abundance is consistently directionally associated with lower methane emissions (Supplementary Table 6).

Variance decomposition from the recursive SEM indicated that direct host genetic effects explained most of the methane phenotypic variance across all populations, whereas microbial-mediated genetic contributions through RS_UBA2 were smaller, though these contributions could not be statistically distinguished from zero based on 95% HPD intervals (Supplementary Table 7). In the Holstein population, direct genetic effects accounted for 0.27 of phenotypic variance, whereas RS mediated contributions remained low. In the Irish crossbred population, direct host genetic effects represented the major component (*h*^2^ = 0.63), with a smaller microbial-mediated contribution (*h*^2^ = 0.05). In Canadian population, microbial-mediated effects remained modest (0.04), with direct host genetic effects explaining 0.03 of the phenotypic variance. These results suggest that RS_UBA2 potentially contributes to methane-associated traits, warranting further investigation.

### Functional predictions

To further strengthen our insight with respect to UBA2810, we incorporated additional functional evidence (for method description see Supplementary Information Methods). Particularly, a complete fumarate reductase module (M00150) supports electron flow toward succinate production, a metabolic route known to divert reducing equivalents away from methanogenesis. We further identified genes associated with anaerobic carbon monoxide dehydrogenase activity (cooS; K00198), together with components of the Wood–Ljungdahl pathway, including folD (K01491) and metF (K00297), as well as a complete RNF ferredoxin:NAD oxidoreductase complex (rnfABCDEG; K03612–K03617). Although the pathway appears incomplete, these genes collectively suggest the potential for ferredoxin-dependent anaerobic electron transfer and alternative electron dissipation pathways linked to reductive carbon metabolism. Additional sulfur assimilation-related genes were also detected, although these pathways appeared incomplete and were therefore not interpreted as evidence for dissimilatory sulfate respiration. Members of the Succinivibrionaceae family have previously been proposed to outcompete methanogens and reduce methane production in marsupial animals through alternative hydrogen utilization pathways [30]. Taken together, these genomic features align with the well-documented capacity of members of the Succinivibrionaceae family to suppress methanogenesis by competing for H₂ and redirecting electron flow toward alternative metabolic sinks, consistent with the patterns observed in our dataset (Supplementary Fig. 14).

## Discussion

In this study, we proposed the term Ruminosignatures to define the existence of microbial groups within bovine ruminal microbiota. The identification of microbial co-abundance groups through NMF represents a significant methodological advance in the study of microbial ecosystems. Unlike traditional dimensionality reduction approaches such as principal component analysis or ruminotypes the NMF-based decomposition provides a parts-based representation that is ecologically relevant. PCA imposes orthogonality constraints among components [31], which may obscure overlapping ecological processes that occur simultaneously in the rumen. Although taxa can contribute to multiple components in both PCA and NMF, NMF differs by imposing non-negativity constraints on both the latent components and their coefficients, producing additive, parts-based representations that capture overlapping co-abundance patterns in a more biologically interpretable manner [13]. The advantage of NMF is that these latent variables can reveal underlying biological processes in a more interpretable way. Samples can be expressed as additive mixtures of multiple communities, reflecting the fact that microbial co-abundance groups often interact rather than exist in isolation. Moreover, unlike PCA, NMF does not require variables to be orthogonal or independent. Instead, it searches for latent co-abundance patterns that capture shared variance across taxa. Each microbial group represents a distinct co-abundance pattern, yet taxa can overlap and covary across samples. This approach also provides comprehensive ecological information, detailing which features are co-abundant within each RS and the relative contribution of each RS within individual samples. This reflects the additive nature of microbial ecosystems, where communities interact and respond collectively to environmental and host-specific factors [13]. Unlike ruminotypes, which assign samples to discrete clusters and summarize dominant community structures, NMF represents samples as mixtures of RS, capturing overlapping microbial memberships and continuous variation in community composition. This enables a more nuanced description of the rumen microbiome whereas complementing clustering-based approaches. This analysis reveals that the cattle ruminal microbiota can be accurately described by combinations of at least 14 RS, which, depending on the cattle population analyzed, capture between 96% and 99% of the variance in the bacterial community. This study demonstrated the heritable nature of RS abundance (${\hat{h}}_{\mathrm{a}}^2$ = 0.09–0.58), supporting a significant influence of host genetics on bacterial community assembly, which aligns with previous reports of low to moderate heritability for core microbiota as well as for some rumen taxa [19, 20, 32–34]. The robustness of this framework was further supported by the integrative cross-cohort analysis performed after adjustment for confounding fixed effects, sequencing depth. This analysis identified 15 Ruminosignatures across the combined dataset and confirmed that most of the dominant microbial structures initially detected in the cohort-specific analyses remained reproducible across populations. Ten of the fourteen previously identified dominant RSs were retained, with *Prevotella*- and *UBA2810*-driven signatures remaining the principal contributors to microbial structure across cohorts. These findings indicate that the core ecological organization captured by NMF is not an artifact of individual datasets but reflects reproducible microbial assemblies that persist across heterogeneous cattle populations.

Suggesting their fundamental role in the microbial ruminal ecosystem, two core microbial RS dominated by *Prevotella* and *UBA2810* genera were consistently identified across 2496 cattle with diverse breeds from five countries. The existence of breed-, diet-, and production system–specific RSs was also observed, reinforcing the influence of diet, production system, and host genetics on the assembly of the ruminal microbiota [20, 35]. For example, RS_SFMI was exclusive to beef cattle, whereas RS_RF16 was specific to Holstein or crossbred populations. The RS profiles observed in the Irish crossbred population exhibited the greatest overlap (six out seven) with those identified in both beef and dairy cattle. This finding reflects the diverse genetic background of the Irish crossbred population and highlights the potential of the NMF-based approach to identify both commons and breed-specific microbial group. This qualitative overlap was further supported by direct comparison of the ***W*** matrices across cohorts, which provided a quantitative measure of RS reproducibility. Several shared RS showed very high similarity across populations, with correlations approaching unity for the *Prevotella*- and *UBA2810*-driven signatures. Moreover, the mean correlations observed across cohort comparisons (*r* = 0.81–0.95) indicate that many microbial assemblies remain highly transferable despite differences in breed composition, feeding systems, and geographical origin. Together, these results demonstrate that the latent factors extracted by NMF capture potentially biologically conserved microbial structures rather than cohort-specific statistical artifacts.

Breed-specific and across-breed associations between RS abundance and key traits, including growth performance, feed efficiency, and CH₄ emissions, were also identified. RS_RF16 displayed a consistent positive association with CH₄ in Holstein cows from France, Italy, and the UK. Among the breed-specific associations, the RS driven by *Sharpea* detected in the Irish crossbred population showed a negative link with CH₄. *Sharpea* has been proposed as keystone genera driving a ruminotype “*S*” negatively associated with CH₄ yields in sheep [17] and was also suggested as a biomarker for low CH_4_ production in cattle [21]. The increased abundance of *Sharpea* in the rumen promotes a fermentation pathway that favors lactate and butyrate production. This shift reduces the availability of hydrogen for methanogens, thereby leading to decreased methane emissions in ruminants [36, 37]. In the Irish crossbred and the French Holstein populations, a positive association between the RS driven by *Ruminococcus* and CH₄ emissions was observed. In contrast to *Sharpea*, an elevated abundance of *Ruminococcus* has been documented in a ruminotype “*H*,” which was linked to higher CH₄ emissions [17]. These findings demonstrate that the RS models confirm key ecological roles known from previous ruminotype concepts while enabling the detection of gradual shifts within the cattle ruminal bacterial community.

Recent findings [38] proposed 14 genera as the core of the ruminant microbiome. These core taxa are characterized by broad metabolic potential, nutritional independence, and extensive redundancy across key biosynthetic pathways. Such functional traits, together with their ability to degrade plant polysaccharides through diverse glycosyl hydrolases and to synthesize essential metabolites such as amino acids and vitamins, highlight their ecological role as keystone taxa within the ruminal ecosystem and lead to the suggestion that these taxa play a central role in supporting host metabolism and ecosystem function [38]. In the present study, we identify Ruminosignatures that consistently covary in abundance across animals, without *a priori* assumptions regarding function, whereas nonetheless showing consistent associations with host traits. Particularly, seven of the core genera reported by Tovar-Herrera *et al*. [38] were among the major drivers of the Ruminosignatures (*Prevotella, Fibrobacter, Succiniclasticum, Ruminococcus, Treponema, Succinivibrio*, and *Butyrivibrio*). Moreover, five of them (*Saccharofermentans, Succiniclasticum, Succinivibrio, Ruminococcus*, and *Prevotella*) play a pivotal role in the assembly of the two core RS identified in our study (Supplementary Fig. 12). This suggests that their influence on community structure is both disproportionate and mechanistically grounded in their metabolic versatility. Such generalism behavior likely contributes to RS assembly and to the metabolic support of non-core taxa, reinforcing their central role in maintaining rumen functional stability, community organization, and host–microbiome symbiosis. Together, these observations suggest that the host-associated patterns captured by RS may, at least in part, stem from the characterized metabolic roles of core taxa described previously by Tovar-Herrera *et al*. [38], reinforcing the view that their contribution to host metabolism is an emergent property of coordinated microbial behavior rather than isolated taxon-specific effects.

To identify microbial groups associated with CH₄ emissions, we tested associations between individual Ruminosignature abundances and CH₄ output, revealing multiple RS linked to methane emissions. However, the direction and strength of this link vary across cohorts (Fig. 5, Supplementary Fig. 13). Among them, despite substantial variation in environmental conditions, farm management practices, host genetic background, and CH₄ measurement methodologies, the abundance of the RS_UBA2 was consistently negatively associated with methane emissions across the full dataset, as well as with the Ace:Prop ratio in the Canadian cohort. Moreover, a positive link of RS_UBA2 with feed efficiency was observed in the Italian Holstein cows and the Irish crossbred populations. The genus *UBA2810* that drives RS_UBA2 was classified within the Gammaproteobacteria and exhibits phylogenetic affinity to the family Succinivibrionaceae. Members of this lineage are known for their ability to produce succinate, primarily through the reduction of fumarate. Succinate production, similar to propionate formation, constitutes a hydrogenotrophic process that can compete directly with methanogens for available hydrogen, thereby limiting methane formation [39]. In fact, several studies have consistently reported an association between members of the Succinivibrionaceae family and CH_4_ production in ruminants and other herbivores [18, 19, 30, 40, 41]. The association of these bacteria with lower methane emissions may be attributed to their hydrogenotrophic metabolism, which limits availability of hydrogen, an essential electron donor in methanogenesis. In agreement with this proposed mechanism, a negative correlation between the abundance of RS_UBA2 and the abundance of the methanogenic archaea *Methanobrevibacter* was observed across all populations (*r*_Holstein_ = −0.39; *r*_Canada_ = −0.33; *r*_Ireland_ = −0.25). Together, these patterns suggest that the host-associated effects captured by this Ruminosignature likely reflect the previously characterized metabolic role of Succinivibrionaceae in hydrogen redirection, reinforcing their contribution to methane mitigation at the level of coordinated microbial behavior, thereby strengthening the interpretation that Ruminosignatures capture metabolically grounded, functionally coordinated microbial behavior linked to host phenotypes. Additionally, in the Holstein discovery population, the abundance of RS_UBA2 showed strong negative correlation (*r* = −0.52, *P* < .001) with the abundance of the holotrich protozoa *Dasytricha*, but displayed a positive relationship with the abundance of *Entodinium* (*r* = 0.51, *P* < .001). *Methanobrevibacter* is the dominant genus of rumen methanogens playing a major role in CH_4_ production via hydrogenotrophic metabolism. *Dasytricha* produces large amounts of hydrogen, which *Methanobrevibacter* use as substrate to generate CH_4_ [42]. In fact, a positive association of *Methanobrevibacter* and *Dasytricha* with CH_4_ and the symbiotic relationship between them are well documented [18, 43–45].

This interpretation was further reinforced by genome-based functional prediction of *UBA2810* sp002351705, which revealed metabolic features consistent with alternative hydrogen utilization and anaerobic electron-transfer pathways. In particular, genes related to fumarate reduction, anaerobic carbon monoxide dehydrogenase activity, partial Wood–Ljungdahl pathway components, and a complete RNF ferredoxin:NAD oxidoreductase complex suggest the potential for redirecting reducing equivalents away from methanogenesis through alternative reductive metabolic pathways. The genomic evidence strengthens the biological plausibility of the observed association between RS_UBA2 abundance and reduced methane emissions. It should be acknowledged, however, that *UBA2810* sp002351705 is currently uncultured and absent from any culture collection, which precludes direct experimental validation of these inferred functions.

Beyond correlation, recursive structural equation modeling provided additional support for a directional effect of RS_UBA2 abundance on methane-related traits. The sign of the structural coefficient was concordant with the sign of the genetic correlation across all three populations (all negative), supporting the expected direction of phenotypic response to selection on RS_UBA2 and addressing concerns about unintended responses to Ruminosignature-based selection strategies. The absence of consistent statistical support across all populations indicates that, although RS_UBA2 was broadly associated with methane-related phenotypes, the strength of the inferred directional effect may have been influenced by differences in genetic background, breed composition, diet, management systems, and effective sample size. Predominantly negative genetic and phenotypic correlations across all populations provide convergent evidence supporting the biological plausibility and consistency of this relationship [46], suggesting that cows with higher RS_UBA2 abundance tend to be genetically associated with lower CH₄ emissions. Variance decomposition further indicated that microbial-mediated genetic effects through RS_UBA2 potentially explained a smaller proportion of methane phenotypic variance relative to direct host genetic effects, supporting the view that this Ruminosignature contributes to methane variation as one component of a broader host–microbiome interaction framework.

In agreement with previous studies [11], the negative association between CH₄ emissions and the abundance of RS_*Prev* was not consistent across populations, which may reflect functional heterogeneity within the *Prevotella* genus. *Prevotella* is a metabolically versatile taxon capable of processing a wide range of proteins and polysaccharides, and one that has been repeatedly associated with low methane emissions in previous studies [34, 47, 48]. *Prevotella* is a well-characterized propionate producer [47], and propionate formation represents a metabolic route in which net hydrogen is consumed rather than released [49]. In addition, *Prevotella* is predicted to produce amino acids such as lysine and cysteine, which can serve as precursors for butyrate and propionate synthesis [49]. Together, these metabolic traits suggest that RS_Prev has the capacity to serve as an alternative and channeling reducing equivalents into alternative fermentation end products. Taken together, these findings demonstrate a coherent association between the increased abundance of RS_UBA2 and context specify for RS_Prev, their predicted hydrogen-consuming metabolic functions, and reduced methane emissions. This convergence of taxonomic identity, functional potential, and phenotypic outcome supports a model in which specific bacterial Ruminosignatures actively redirect electrons flow away from methanogenesis, contributing to lower methane production in the rumen ecosystem.

Some limitations should be considered in this study. The dynamic nature of microbial groups which shift with host age has been documented in human and pig gut microbial ecosystems [13, 15]. The current study focused exclusively on adult animals; it overlooked the age-related dynamics of microbial group assembly and potentially missed critical insights into early-life development. This is particularly relevant for putative dietary interventions to modulate RS abundance given that early life has been proposed as a window of opportunity for modulating the ruminal microbiota [50–52]. Moreover, these findings are based solely on metataxonomic information, which limits the ability to draw conclusions about the functional roles of RS. Therefore, to capture active microbial processes and gain a more comprehensive understanding, future studies should incorporate functional approaches from other source of information such as metatranscriptomics and metaproteomics. Finally, we acknowledge that genus-level aggregation can mask strain or species level heterogeneity, merge organisms with distinct ecological roles, and implicitly assume ecological coherence within genera. Consequently, NMF-derived RS should be interpreted as genus-level co-abundance structures that likely capture shared ecological organization, while acknowledging that additional taxonomic resolution may further refine their functional interpretation.

Despite these limitations, the findings herein offer novel insights into the assembly of cattle ruminal microbiota. This study demonstrated that RS are heritable and associated with key traits in cattle. Notably, RS_UBA2 exhibited sufficient stability across multiple breeds and different environments to be considered a promising candidate proxy for potential integration into breeding programs aimed at mitigating CH_4_ emissions. Such proxies could reduce the need for long and costly CH_4_ measurement and potentially enhance the accuracy of selection when combined with host genetic information. The practical relevance of RS_UBA2 was further strengthened by meta-analysis across countries, which demonstrated highly consistent associations with methane-related traits, acetate ratio, and average daily gain after multiple-testing correction. Collectively, the opposite significant directions observed for ADG relative to methane-related traits, together with the negative association with FCR, suggest that increased RS_UBA2 abundance may be linked to improved animal efficiency and reduced CH₄ emissions. Furthermore, a strong negative association between the Ace:Prop ratio and the abundance of ASV assigned to the genus UBA2810 was reported in a Holstein population [53]. In addition, UBA2810 was identified as a key microbial signature associated with feed efficiency in growing pigs [15], further supporting its potential involvement in host metabolic efficiency. Collectively, these findings highlight the relevance of *UBA2810* beyond a single host species and suggest a potential contribution to livestock metabolic efficiency.

Microbial traits can be integrated into genetic evaluations either as additional traits that are genetically correlated with host traits, improving prediction accuracy, or by using microbial profiles to construct relationship matrices that explicitly capture host microbiome interactions [7]. The implementation of NMF to infer microbial groups represents a step forward toward ecosystem-based modeling of the rumen microbiome, supporting the idea that ecosystem-level interactions, rather than individual microorganisms, underlie key ruminal processes influencing host productivity and environmental impact. This approach facilitates the integration of microbial community structure with host and environmental variables, opening avenues for predictive modeling, causal inference, and the discovery and design of synthetic consortia for targeted microbial modulation in livestock. An additional strength of the present framework is that the reference ***W*** matrix and corresponding Ruminosignatures definitions are publicly available, enabling direct projection of independent datasets onto a common microbial reference space without repeating the full matrix factorization procedure. This substantially improves cross-study comparability and facilitates external validation, addressing one of the major limitations in rumen microbiome research where differences in sequencing pipelines and cohort composition often hinder reproducibility.

## Conclusions

This study provides new insights into the assembly of the cattle rumen microbiota by introducing the Ruminosignatures concept. Common and population-specific heritable microbial groups associated with CH₄ emissions and feed efficiency were identified. These findings suggest that classifying rumen microbiota into RS could play a key role in microbiome-informed precision farming and breeding programs aimed at reducing the cost of feeding animals and lowering the environmental footprint of cattle production. Yet, there are particularities associated with the breed, diet, and production system that emphasize the importance of tailoring modulatory strategies to specific breeding and production systems.

## Supplementary Material

Supplementary_Information_final_updated_wrag185

## Data Availability

The raw sequencing data employed in this article has been submitted to the NCBI’s Sequence Read Archive (https://www.ncbi.nlm.nih.gov/sra) and BioProject under accession numbers PRJNA517480, PRJNA797238, PRJNA393057, and PRJEB98694. All the input data used in the analysis are available in the Zenodo repository (https://doi.org/10.5281/zenodo.19709125).
