# Supplementary material for: Ruminosignatures associated with methane emissions and feed efficiency across geographies and cattle breeds
Source: ISME J. 2026 Jul 12;20(1):wrag185. doi: 10.1093/ismejo/wrag185 (PMC13573583; doi:10.1093/ismejo/wrag185)
Supplement: Supplementary file 1 [file supplementary_information_final_updated_wrag185.pdf]

# Ruminosignatures associated with methane emissions and feed efficiency across geographies and cattle breeds

Ioanna-Theoni Vourlaki[1], Ori Furman[2], Ilma Tapio[3], Le Luo Guan[4], Sinéad M. Waters[5], David Kenny[6] , Paul Smith[6], Stuart F. Kirwan[7], David Kelly [8], Ross Evans [8] , Raquel Quintanilla[1], Miriam Piles[1], Antonio Reverter [9] , Pâmela A. Alexandre [9], Fuyong Li[10], Philip C. Garnsworthy[11], Paolo Bani[12] , Phillip B. Pope[13,14], Diego P. Morgavi[15], Itzhak Mizrahi[2] , Yuliaxis Ramayo-Caldas[1]

[1]Animal Breeding and Genetics Program, IRTA, Torre Marimon, 08140 Caldes de Montbui, Spain, [2] Department of Life Sciences, Ben-Gurion University of the Negev, Beer-Sheva 84105, Israel, [3] Genomics and Breeding, Natural Resources Finland (Luke), Jokioinen 31600, Finland, [4] Faculty of Land and Food Systems, The University of British Columbia, V6T 1Z4 Vancouver, Canada, [5] School of Biological and Chemical Sciences and Ryan Institute, University of Galway, Galway H91 TK33, Ireland, [6] Animal and Bioscience Department, Teagasc Grange, Dunsany, Co. Meath C15 PW93, Ireland, [7] Department of Animal Science, University of California, Davis, CA 95616, United States, [8] Irish Cattle Breeding Federation, Link Road, Ballincolling, Co. Cork, P31 D452, Ireland,[9] CSIRO Agriculture and Food, St. Lucia, Brisbane, QLD 4067, Australia,[10] Department of Animal Science and Technology, College of Animal Sciences, Zhejiang University, Hangzhou, 310058, PR China,[11] School of Biosciences, University of Nottingham, Sutton Bonington Campus, Loughborough LE12 5RD, United Kingdom,[12] Department of Animal Science, Food and Nutrition, Università Cattolica del Sacro Cuore, 29122 Piacenza, Italy,[13] Centre for Microbiome Research, School of Biomedical Sciences, Queensland University of Technology (QUT), Translational Research Institute, Woolloongabba, QLD, 4102, Australia,[14] Faculty of Chemistry, Biotechnology and Food Science, Norwegian University of Life Sciences, 1432 Ås, Norway,[15] Université Clermont Auvergne, INRAE, VetAgro Sup, UMR Herbivores, Saint-Genès-Champanelle, F-63122, France

## Methods

### Association of Ruminosignatures with methane emission and feed efficiency

The association analyses were performed using a univariate linear model approach, applied separately to each of the datasets included in this study. For each analysis, the phenotype of interest (methane emission or feed efficiency trait) was included as the dependent variable, whereas the abundance of a single Ruminosignature (RS) was fitted as the predictor of interest. All models were fitted in R [1] using the *lm* function, and the results were visualized with the *ggplot2* [2] package. Given the differences across datasets in both structure and available metadata, dataset-specific covariates were included in the models as described below. Thus, the reported associations correspond to the estimated regression coefficient of RS abundance, and the reported *P* values were obtained from a two-sided test of the null hypothesis that the regression coefficient of the RS abundance was equal to zero ($H_0$: $\beta RS = 0$). *P* values < 0.05 were considered significant.

In the Holstein discovery dataset, we assessed the links between RS abundance and $CH_4$ and feed efficiency-related traits, including $CH_4$ yield per day ($CH_4$ g/d), $CH_4$ yield per kilogram dry matter intake ($CH_4$ g/kg DMI), energy-corrected milk to DMI ratio (ECM kg/DMI), and residual feed intake based on NRC (RFI.NRC). Analyses were performed by country: Italy (IT) and the UK, incorporating the first five principal components to control for population structure. Farm of origin was included as a fixed effect (IT three levels and UK two levels). In the French validation dataset, RS associations with $CH_4$ g/d were assessed in the subset 65 cows with available measurements. Because $CH_4$ were not directly measured in the Canadian dataset, we used the acetate-to-propionate ratio (Ace: Prop) as a proxy for $CH_4$ production. RS abundance was tested for association with Ace: Prop and additional performance traits including ADG, RFI, and FCR. Analyses were conducted separately by breed: Angus (ANG), Charolais (CHAR), and Kinsella composite hybrid (HYB). All traits were corrected for age, whereas sex (two levels: females, N = 347; males, N = 358) and diet (four levels) were included as fixed effects. Finally, for crossbred Ireland cohort, associations between RS abundance and FCR, ADG, $CH_4$ g/d, and $CH_4$ g/kg DMI were evaluated. Models included age, breed, contemporary group, and the first five principal components as covariates, whereas sex (two levels: females = 352; males = 532) and dam parity (5 levels) were included as fixed effects. To account for breed composition in a crossbred cattle population, we constructed a categorical breed variable based on genomic breed

fractions using discriminant analysis of principal components (see Supplementary Information Methods section "Construction of a categorical breed variable using DAPC").

**Construction of a categorical breed variable using DAPC**

To account for breed composition in a crossbred cattle population, we constructed a categorical breed variable based on genomic breed fractions. The aim was to reduce dimensionality while preserving the main breed structure in the population for inclusion as a fixed effect in the association analysis, heritability estimation and structural equation model (SEM). Each animal had breed fractions for multiple breeds (e.g., HO, FR, AA, LM, CH, etc.), where the fractions represent the proportional genetic contribution of each breed and sum to 1 for every individual. We used discriminant analysis of principal components (DAPC, [3]), that is a multivariate method designed to investigate the genetic structure of biological populations. We used *adegenet* [4] package in R. First, we identified clusters using the find.clusters() function. The optimal number of clusters (k) was determined based on the decrease in the Bayesian Information Criterion (BIC) which suggested five clusters in the population. Next, we applied DAPC to efficiently describe the genetic clusters using a few synthetic variables. These variables are constructed as linear combinations of the original variables (alleles) that maximize between-group variance and minimize within-group variance. The coefficients of the alleles in these linear combinations are referred to as loadings, and the resulting synthetic variables are called discriminant functions. DAPC also provides membership probabilities of each individual for the different clusters based on the retained discriminant functions. These probabilities indicate the clarity of genetic clustering: loose clusters result in flat distributions of membership probabilities across clusters, suggesting potential admixture. Based on the final DAPC cluster assignments, the categorical breed variable for inclusion in the SEM was defined, capturing the main breed structure of the population.

**Host genotype and heritability estimation**

Cows from the Holstein discovery dataset were genotyped with the Bovine GGP HD chip v2 that included 138,892 SNP, the Irish crossbred were genotyped with a custom chip (International Dairy and Beef Chip Illumina BeadChip), latter imputed to a 50K SNPs using Fimpute [5], whereas the other populations were genotyped with the Illumina BovineSNP50 v2 Genotyping BeadChip which contained 54,609 SNP (San Diego, CA, USA). Plink software [6] was used to remove SNPs that had a minor allele

frequency less than 5%, that had more than 10% missing genotype data, mapping to the sex chromosome, or that did not map to the ARS-UCD1.3 bovine assembly. SNP genomic positions were verified by retrieving the rsIDs for each SNP and extracting their corresponding genomic coordinates directly from the ARS-UCD1.3 reference genome.

The potential host genetic control on RS abundance was explored by estimating the genetic variance components. Specifically, for each of the datasets, the narrow-sense heritability ($\hat{h}_a^2$) was estimated by implementing the Reproducing Kernel Hilbert Space (RKHS, [7]) model from the BGLR package [8] applied separately to each cohort. Prior to analysis, the abundance of each RS was subjected to an inverse normal (rank-based) transformation to ensure homoscedasticity in the model residuals.

After quality control the genotypic data for the Holstein discovery set included 109,193 SNP across 771 animals. The model was implemented jointly for both countries (IT and UK), with farm included as a fixed effect as follows:

$$\mathbf{y}_{\mathbf{RS}} = \mathbf{X\beta} + \mathbf{Zu}_{\boldsymbol{Holstein}} + \mathbf{e}$$

where $\mathbf{y}$ is the vector of individual RS abundances, $\boldsymbol{\beta}$ is the vector of the systematic effects of farm (five levels) and $\mathbf{X}$ its corresponding incidence matrix, $u$ is the vector of additive genetic random effects, whereas $\mathbf{e}$ is the vector of residuals.

The Canadian dataset comprised genotypic information for 43,123 SNP across 685 samples, whereas the Irish dataset included 41,335 SNPs from 843 individuals. The mixed model was applied independently to each dataset, as described below:

$$\mathbf{y}_{\mathbf{RS}} = \mathbf{X\beta} + \mathbf{Zu}_{\boldsymbol{Canada}} + \mathbf{e},$$

where, β is the vector of the covariate age and fixed effects of sex (two levels), breed (three levels), diet (four levels), and $\mathbf{X}$ their corresponding incidence matrix.

$$\mathbf{y}_{\mathbf{RS}} = \mathbf{X\beta} + \mathbf{Zu}_{\boldsymbol{Ireland}} + \mathbf{e},$$

where, β is the vector of the covariate age, contemporary group and systematic effects of sex (two levels) and dataset (two levels), dam parity (5 levels) and breed (5 levels). In all datasets random genetic effects were assumed to follow a multivariate normal distribution $\mathbf{u} \sim N(0, \boldsymbol{G}\sigma_a^2)$, where $\mathbf{G}$ is the genomic relationship matrix (GRM) calculated using VanRaden method and $\sigma_a^2$ is the additive genetic variance. The GRM was obtained as $\boldsymbol{G} = \frac{\boldsymbol{ZZ'}}{2\sum_j p_j(1-p_j)}$, here $p_J$ is the minor allele frequency of SNP *j,* and *Z*

is the MAF-adjusted genotype matrix generated with the AGHMatrix package [9, 10]. For the estimation of genetic variances with BGLR, default priors were used. Thus, the narrow-sense heritability is defined as the proportion of RS variance explained by additive genetic effects as follows:

$$\mathbf{\hat{h}_a^2} = \mathbf{s_a^2}/(\mathbf{s_a^2} + \mathbf{s_e^2})$$

where $\mathbf{s_a^2}$ and $\mathbf{s_e^2}$ are the posterior estimates of the additive genetic and residual variances respectively. **BGLR** was run for 100,000 iterations with a burn-in of 500 and thinning interval of five. Convergence was assessed visually, and the corresponding plots are provided in Supplementary Figures 1-3 for each dataset. Posterior Gibbs samples representing $\mathbf{\hat{h}_a^2}$, were entered into a Monte Carlo Markov Chain (MCMC) object using the as.mcmc() function from the coda package in R [11] whereas the 95% highest posterior density intervals (HPD95) were computed for each heritability estimate.

**Integrative cross-cohort analysis methodology**

We extended the Ruminosignature framework by performing an integrative cross-cohort analysis across all available countries to evaluate whether the microbial signatures identified in the cohort-specific analyses were globally consistent or cohort-dependent. Particularly, to account for confounding fixed effects across these heterogenous datasets while maintaining the non-negativity required for downstream Non-negative Matrix Factorization (NMF), we implemented a multiplicative adjustment strategy based on marginal standardized predictions. Firstly, for each dataset we kept genera present in at least 5% of samples. Then we kept genera that appear in at least three out of the four datasets. This resulted in 59 genera across all datasets. For each of the 59 genera, a Generalized Linear Model (GLM) was fitted using either a Negative Binomial or Poisson distribution (as a fallback for non-converging models) to model the raw counts as a function of Sex, Breed, and Country, with the logarithm of sequencing depth included as an offset. To remove the influence of these covariates, we calculated two predicted values for every sample: the actual predicted mean $\hat{y}_{actual}$ based on the observed metadata, and a reference predicted mean $\hat{y}_{ref}$ obtained by setting all categorical predictors to the most frequent combination in the dataset and the offset to the global mean. The corrected counts were then derived by multiplying the observed counts by the ratio of the reference prediction to the actual prediction $y_{corr} = y_{obs} \cdot \frac{\hat{y}_{ref}}{\hat{y}_{actual+\epsilon}}$, where ∈ is a small constant

($10^{-8}$) to avoid division by zero. This ratio-based approach effectively "neutralizes" the systematic bias attributed to fixed effects while preserving individual-level biological variation and ensuring all values remained non-negative. Finally, the corrected counts were normalized using Total Sum Scaling (TSS) to represent genus relative abundances for NMF input. The effectiveness of the correction was validated using Permutational Multivariate Analysis of Variance (PERMANOVA). The total variance explained by the confounding factors (Breed, Sex, Country, and sequencing depth) was reduced from 40.4% in the raw relative abundance data to 17.3% in the corrected matrix, successfully increasing the residual biological variation available for NMF signature extraction. That means that we increased the Residual variance from 59.6% to 82.7%, unmasking the individual biological variation for NMF. PERMANOVA was performed using the adonis2() function from the vegan R package [12], based on Bray-Curtis dissimilarity metric with 99 permutations.

**Comparative analysis of taxonomy contribution across core Ruminosignatures**

To gain deeper insight into RS assembly and the putative ecological roles of individual genera within the core microbial co-abundance groups (RS_Prev and RS_UBA2), we performed a stratified analysis by dataset. For each dataset, genera with a non-zero relative contribution, based on the normalized W matrix, in at least one RS were retained. Then, the different datasets were intersected, and the genera shared across all of them were identified. Supplementary Figure 12 illustrates the fourteen genera commonly associated with at least one of the core groups across all cattle populations, revealing distinct yet recurrent patterns. As previously described, the core-RS were consistently dominated by *Prevotella* and *UBA2810*, that contributed the largest proportion of the RS community structure. Remarkably, despite differences in diet, genetic background, farm of origin, country or production system, the identity of the dominant genus remained consistent across all datasets. However, the relative contribution of the dominant genus varied in magnitude; for instance, *UBA2810* exhibited a higher contribution in the Canadian beef cohort (90%) and a lower (60%) in the Irish crossbred. Beyond the dominant genera, we also observed consistent trends among less prevalent genera that contribute to the composition of RS_Prev and RS_UBA2. For example, *Cryptobacteroides* appeared consistently linked to RS_Prev, with comparable contributions across datasets. Similarly, *Paraprevotella* exhibited strong co-occurrence patterns to RS_Prev across datasets. Notably, RF16 was detected exclusively in RS_Prev,

whereas genera such as *Ruminococcus_E* and *Succinivibrio* displayed a marked shift in abundance depending on the RS type. CAG-269 followed a similar pattern in both RSs, although its highest contribution was observed in RS_UBA2. Overall, RS_Prev is robustly defined by *Prevotella*, while the presence and abundance of co-occurring genera appear to be context dependent. Likewise, RS_UBA2 was consistently drived by *UBA2810*, though the composition of associated genera varies considerably between populations. While RS type remains a major driver of genus-level structure, population-specific factors also modulate the contribution of individual genera. RS_Prev and RS_UBA2 harbor distinct microbial signatures: some genera are consistently enriched in one RS across all datasets, serving as robust markers of RS identity, whereas others exhibit variable patterns, likely influenced by host or environmental context.

**Pairwise cross-cohort similarity analysis**

To assess the reproducibility and transferability of RS across different cattle populations, we compared the ***W*** matrices obtained from NMF for each cohort. In these matrices, columns correspond to RS and rows correspond to genera, with values representing the contribution of each genus to the corresponding RS. For each pair of cohorts, we then computed the Pearson correlation between all RS from cohort 1 and all RS from cohort 2. Then, for each RS in cohort 1, the highest correlation coefficient was retained to identify the most similar RS in cohort 2. The resulting table includes the correlation coefficient, providing a quantitative measure of cross-cohort similarity. To visualize similarity patterns, correlation matrices were displayed as heatmaps, where rows correspond to RS from cohort 1, columns correspond to RS from cohort 2, and cell colours represent the correlation strength.

**Meta-analysis**

A meta-analysis was performed to summarize the associations of RS_UBA2 with feed efficiency and methane-related traits across multiple countries based on a weighted, sign-aware Stouffer meta-analysis based on two-sided p-values. Individual country-specific p-values were capped at $2\times10^{-15}$ to avoid computational issues arising from extremely small values, then transformed to Z-scores using the inverse normal method. Each Z-score was weighted by the square root of the corresponding sample size, and the weighted Z-scores were aggregated to produce a combined test statistic. The direction of association was determined from the weighted combined Z-score, accounting for both sample size and effect direction. Multiple testing across traits was addressed using both FDR (Benjamini–Hochberg) and Bonferroni corrections (Supplementary Table 5).

## Structural equation modelling for causal inference

To distinguish microbial biomarkers from potential causal mediators, we implemented a bivariate recursive structural equation modelling framework that jointly estimated the recursive effect of RS abundance on methane-related traits together with the genetic correlation between both traits, following the model structure described in [13].

Specifically, structural equation models with recursive effects were applied separately to each population to evaluate the genetic relationship between methane traits and RS associated with UBA2810. In addition, structural coefficients were estimated for the direct effect of RS_UBA2 on methane traits in order to quantify the strength of this association. The corresponding results are presented in Supplementary Tables 6 & 7. Our objective was to quantify the extent to which RS driven by UBA2810 mediates methane emissions across datasets. For this purpose, we implemented a recursive model in which the individual RS_UBA2 abundance (RS) was fitted as a potential mediator of methane traits (M). In this framework, the host genome (G) influences both the phenotype (G→M) and the rumen signature (G→RS), while the microbiome also affects the phenotype through the pathway RS→M, thereby representing a mediator of indirect genetic effects (G→RS→M). The structural coefficient ($\lambda_{M\leftarrow RS}$) quantifies the causal effect of $RS$ on $M$, while conditioning on G. Additionally, dataset-specific environmental factors ($E_M$ $and$ $E_{RS}$) were included to independently account for environmental sources of variation affecting $RS$ and $M$, respectively assuming no correlation between these effects. The recursive models were formulated as bivariate models, in which one methane-related trait and one RS associated with UBA2810 were analyzed jointly and separately for each population, as follows:

$$\Lambda y = Wb + g + e,$$

where, $y$ is the vector of phenotypic records for M ($CH_4$ g/d, Ace: Prop) and RS the RS_UBA2810; $\Lambda$ is the matrix of structural coefficients for RS: $\Lambda = \begin{bmatrix} 1 & 0 \\ -\lambda_{M\leftarrow RS} & 1 \end{bmatrix}$, where $\lambda_{M\leftarrow RS}$ is the effect of RS on M; $b$ is the vector of fixed effects for each of datasets. For the Canada dataset, the model included sex, diet, and breed as fixed effects, with age included as a covariate. For the Holstein dataset, the model included farm, country, and methane emission measurement method as fixed effects. For the

Ireland dataset, the model included dataset, contemporary group, sex, dam parity, and breed as fixed effects.

The recursive model assumes that genomic and residual effects follow multivariate normal distributions such that $g \sim N(0, G_0 \otimes G)$ and $e \sim N(0, H_0 \otimes I_n)$, where G is the genomic relationship matrix and $I_n$ is an identity matrix of order $n$. The matrices $G_0$ and $H_0$ denote the symmetric covariance matrices for genomic and residual effects, respectively across the two traits (M and RS), with their elements defined as follows:

$$G_0 = \begin{bmatrix} \sigma^2_{g,M} & \sigma_{g,MRS} \\ symmetric & \sigma^2_{g,RS} \end{bmatrix} \text{ and } H_0 = \begin{bmatrix} \sigma^2_{e,M} & \sigma_{e,MRS} \\ symmetric & \sigma^2_{e,RS} \end{bmatrix}$$

Where $\sigma^2_{g,M}$ and $\sigma^2_{g,RS}$ correspond to the additive genetic variances for the phenotype and the microbiome-related variable, respectively. The term $\sigma_{g,MRS}$ represents the genetic covariance between both traits and reflects the extent to which common genetic determinants contribute to variation in the phenotype and microbial trait, potentially due to pleiotropic effects or linkage disequilibrium. Similarly, $\sigma^2_{e,M}$ and $\sigma^2_{e,RS}$ denote the residual variances associated with the phenotype and microbiome, whereas $\sigma_{e,MRS}$ describes the residual covariance between them. To ensure parameter identifiability in the recursive SEM, $H_0$ was constrained to a diagonal structure, which assumes absence of residual covariance between traits $\sigma_{e,MRS} = 0$. Under this specification the recursive model can be parameterised as a standard mixed model by multiplying all terms by $\Lambda^{-1}$, as follows:

$y = \Lambda^{-1}Wb + \Lambda^{-1}g + \Lambda^{-1}e = \Lambda^{-1}Wb + g^* + e^*,$ where, $g^*$ and $e^*$ represent the vectors of breeding values and residuals under the standard mixed model, and $G_0^* = \Lambda^{-1}G_0\Lambda^{-1'}$ and $H_0^* = \Lambda^{-1}H_0\Lambda^{-1'}$ [14 ]:

$$G_0^* = \begin{bmatrix} \sigma^2_{g*,M} & \sigma_{g*,MRS} \\ \sigma_{g*,MRS} & \sigma^2_{g*,RS} \end{bmatrix} \text{ and } H_0^* = \begin{bmatrix} \sigma^2_{e*,M} & 0 \\ 0 & \sigma^2_{e*,RS} \end{bmatrix}$$

In the recursive formulation, the vector $g$ is partitioned into two components: $g_M$, which represents the direct additive genetic effect and $g_{RS}$ which captures the ruminosignature effect. Furthermore, $\lambda_{M \leftarrow RS} g_{RS}$ represent the individual genomic effects that are mediated by RS on M, such that $g^*_M = g_M + \lambda_{M \leftarrow RS} g_{RS}$, is the overall host genetic effects on the phenotype after accounting for both direct and microbiome-mediated pathways. Under this formulation, the components of $G_0^*$ are defined as follows:

$$\sigma^2_{g*M} = \sigma^2_{g,M} + 2\lambda_{M\leftarrow RS}\sigma_{g,MRS} + \lambda^2_{M\leftarrow RS}\sigma^2_{g,RS}$$

$$\sigma^2_{g*RS} = \sigma^2_{g,RS}$$

$$\sigma_{g*,MRS} = \sigma_{g,MRS} + \lambda_{M\leftarrow RS}\sigma^2_{g,RS}$$

Where, $\sigma^2_{g*M}$ and $\sigma^2_{g*RS}$ denote the total genetic variances associated with the phenotype and the ruminosignature, respectively. Under this parameterization, the corresponding expression also defines the genetic covariance between both traits after accounting for the recursive relationship. An important implication is that when $\lambda_{M\leftarrow RS}\sigma^2_{g,RS}$ has the same magnitude but opposite sign as $\sigma_{g,MRS}$, the resulting total genetic covariance becomes zero. Assuming Gaussian-distributed effects, this condition indicates statistical independence between the genetic components affecting the two traits. Recursive models were fitted using the BLUPF90 family of programs [15] under a Bayesian framework via Gibbs sampling using the Gibbsf90 software with a single Markov chain of 100,000 iterations, including 10,000 burn-in samples and a thinning interval of 5. Posterior samples of variance components were extracted using the Postgibbsf90 software and subsequently processed in R for variance decomposition and convergence diagnostics. Statistical support for each structural coefficient was assessed using the ratio between the posterior mean and its posterior standard deviation, $Z = \frac{\text{mean}(\lambda)}{\text{SD}(\lambda)}$. Structural coefficients were considered significantly different from zero when $|Z| > 2$, corresponding approximately to a 95% confidence level. Convergence of the Markov chains was assessed using the Geweke diagnostic [16], implemented in the coda package in R, for every element of the genetic covariance matrix and for the structural coefficient across all populations. Geweke z-scores compare the posterior means of the first 10% and last 50% of the retained chain; absolute values below 2 indicate satisfactory convergence of the Markov chain.

**Phenotypic Correlation**

Phenotypic correlations between RS_UBA2 and methane emissions were assessed using Pearson's correlation coefficient and are reported in Supplementary Table 6.

**Functional Predictions**

To further strengthen our insight with respect to UBA2810, we incorporated additional functional evidence as follows: First, we identified the target species of the genus

UBA2810 in our metataxonomic data, which was subsequently matched to the species UBA2810 sp002351705 in the Genome Taxonomy Database (GTDB) [17]. Second, the genome of the selected species was retrieved from a GTDB-affiliated repository, specifically the National Center for Biotechnology Information database (GenBank Assembly Accession: GCA_002351705.1). Third, genes were predicted and functionally annotated using DRAM [18] against the PFAM-A, KOfam, and dbCAN-V10 databases, after which KEGG functional module completeness was assessed with MetQy [19] to evaluate the metabolic potential of this genome with respect to $H_2$ utilization and putative competition with methanogenic archaea.

**Supplementary Table 1**. Summary of the employed datasets including country, breed, number of samples and source.

| **Country** | **Breed (type)** | **No samples** | **Source (references)** |
|---|---|---|---|
| United Kingdom | Holstein (dairy) | 409 | *Wallace et al., 2019 [19]* |
| Italy | Holstein (dairy) | 409 | *Wallace et al., 2019 [19]* |
| France | Holstein (dairy) | 65 | *Ramayo-Caldas et al., 2019 [18]* |
| France | Holstein (dairy) | 55 | this study |
| Canada | Angus (beef) | 203 | *Li et al., 2019 [20]* |
| Canada | Charolais (beef) | 114 | *Li et al., 2019 [20]* |
| Canada | Kinsella composite (beef hybrid) | 392 | *Li et al., 2019 [20]* |
| Ireland | Crossbred (beef x dairy) | 272 | *Smith et al, 2022 [21]* |
| Ireland | Crossbred (beef x dairy) | 577 | this study |

**Supplementary Table 2:** Sequencing-depth statistics for each cohort, including minimum, first quartile, median, third quartile, maximum, and mean read frequency per sample.

| Dataset | Statistic | Frequency |
|---|---|---|
| Holstein Discovery | Minimum frequency | 7049 |
| | 1st quartile | 19977 |
| | Median frequency | 33483 |
| | 3rd quartile | 47729.75 |
| | Maximum frequency | 99779 |

| | | |
|---|---|---|
| | Mean frequency | 35150.291 |
| Holstein Validation | Minimum frequency | 16474 |
| | 1st quartile | 24771 |
| | Median frequency | 30876 |
| | 3rd quartile | 34064 |
| | Maximum frequency | 59248 |
| | Mean frequency | 30284.36 |
| Canada | Minimum frequency | 6029 |
| | 1st quartile | 16272 |
| | Median frequency | 20385 |
| | 3rd quartile | 24798 |
| | Maximum frequency | 53915 |
| | Mean frequency | 20811.9 |
| Ireland | Minimum frequency | 20226 |
| | 1st quartile | 34402 |
| | Median frequency | 38801 |
| | 3rd quartile | 47444.5 |
| | Maximum frequency | 135980 |
| | Mean frequency | 45050.1 |

**Supplementary Table 3:** L1 regularization parameter (α) value selected according to the rank considered optimal.

| Country | Best rank | alpha value |
|---|---|---|
| Holstein (discovery set) | 6 | 0.062 |
| Holstein (validation set) | 7 | 0.031 |
| Ireland | 7 | 0.125 |
| Canada | 8 | 0.250 |

**Supplementary Table 4:** Cosine similarity and explained variance scores obtained from NMF decomposition of the whole dataset for the optimal rank.

| Country | Best rank | Cosine similarity | Explained variance |
|---|---|---|---|
| Holstein (discovery set) | 6 | 0.995 | 0.989 |
| Holstein (validation set) | 7 | 0.999 | 0.998 |
| Canada | 8 | 0.979 | 0.958 |
| Ireland | 7 | 0.985 | 0.971 |

**Supplementary Table 5:** Meta-analysis of the association between RS_UBA2810 and production, feed efficiency, and methane-related traits across cohorts. Combined p-values were obtained using a weighted Z-score approach, in which country-specific p-values were transformed into Z-scores and weighted by the square root of sample size prior to aggregation. *Direction* indicates the overall direction of association based on the sign of the combined weighted Z-score. *z* represents the combined test statistic, *P_combined* the meta-analysis p-value, *log10p* the -log10 transformed *P* value, *P_adjusted_fdr* the Benjamini–Hochberg adjusted *P* value, and *P_adjusted_bonf* the Bonferroni-adjusted *P* value. Significance levels are indicated as: *P* < 0.05 *(), P < 0.01 (), and P < 0.001 ()*.

| **trait** | **direction** | **z** | ***P*_combined** | **log10p** | **signif** | ***P*_adjusted_fdr** | ***P*_adjusted_bonf** |
|---|---|---|---|---|---|---|---|
| **ADG** | positive | 7.611 | 2.71E-14 | 13.567 | *** | 4.74E-14 | 1.90E-13 |
| **Ace: Prop** | negative | -8.963 | 3.14E-19 | 18.503 | *** | 7.33E-19 | 2.20E-18 |
| **CH4 g/d** | negative | -11.167 | 5.92E-29 | 28.228 | *** | 2.17E-28 | 4.14E-28 |
| **CH4 g/kg DMI** | negative | -11.163 | 6.21E-29 | 28.207 | *** | 2.17E-28 | 4.35E-28 |
| **ECM kg/DMI** | positive | 1.075 | 0.282 | 0.549 | | 0.282 | 1 |
| **FCR** | negative | -3.116 | 0.002 | 2.737 | ** | 0.0026 | 0.0128 |
| **RFI.NRC** | positive | 1.200 | 0.230 | 0.638 | | 0.268 | 1 |

**Supplementary Table 6:** Phenotypic and genetic correlations between RS_UBA2 and CH4gd or Proxy Ace: Prop, statistical support for the recursive structural coefficient (direct effect), and Geweke convergence diagnostics for the genetic correlation and structural coefficient across cattle populations.

| | Phenotypic Correlation | Genetic Correlation ($r_g$) | Posterior Z ratio (mean/SD) = | Geweke diagnostic (Z-score) |
|---|---|---|---|---|

| | | HPD95 [lower upper] | $\frac{\text{mean}(\lambda_{M<-RS})}{\text{SD}(\lambda_{M<-RS})}$, mean($\lambda_{M<-RS}$), HPD95 [lower upper] | Genetic Correlation | Direct Effect |
|---|---|---|---|---|---|
| HOLSTEIN | -0.34 | -0.49[-0.92,-0.04] | -1.99, -0.03[-0.06, 0.00] | -1.09 | 0.59 |
| IRELAND | -0.52 | -0.65[-0.94,-0.36] | -0.92, -0.08[-0.24,0.09] | 0.09 | -1.13 |
| CANADA | -0.47 | -0.40[-1.00, 0.90] | -12.66. -0.46[-0.52,-0.39] | -0.98 | 1.99 |

Z = posterior mean divided by the posterior standard deviation of the structural coefficient ($\lambda_{M \leftarrow RS}$); larger absolute values indicate stronger statistical support for the direct effect.
and
Geweke diagnostic = MCMC convergence diagnostic. Absolute Z-scores close to zero indicate satisfactory convergence of the Markov chain.

**Supplementary Table 7:** Contribution of direct and microbial-mediated genetic effects to the phenotypic variance

| | **phenotypic variance microbial-mediated genetic effects** | | **phenotypic variance of direct effects** | |
|---|---|---|---|---|
| | HPD95 [lower upper] | Mean $h^2_{M<-RS}$ | HPD95 [lower upper] | Mean $h^2{}_M$ |
| HOLSTEIN | [0.00, 0.23] | 0.01 | [0.12,0.43] | 0.27 |
| IRELAND | [0.00, 0.10] | 0.05 | [0.44, 0.83] | 0.63 |
| CANADA | [0.00, 0.09] | 0.04 | [0.00, 0.07] | 0.03 |

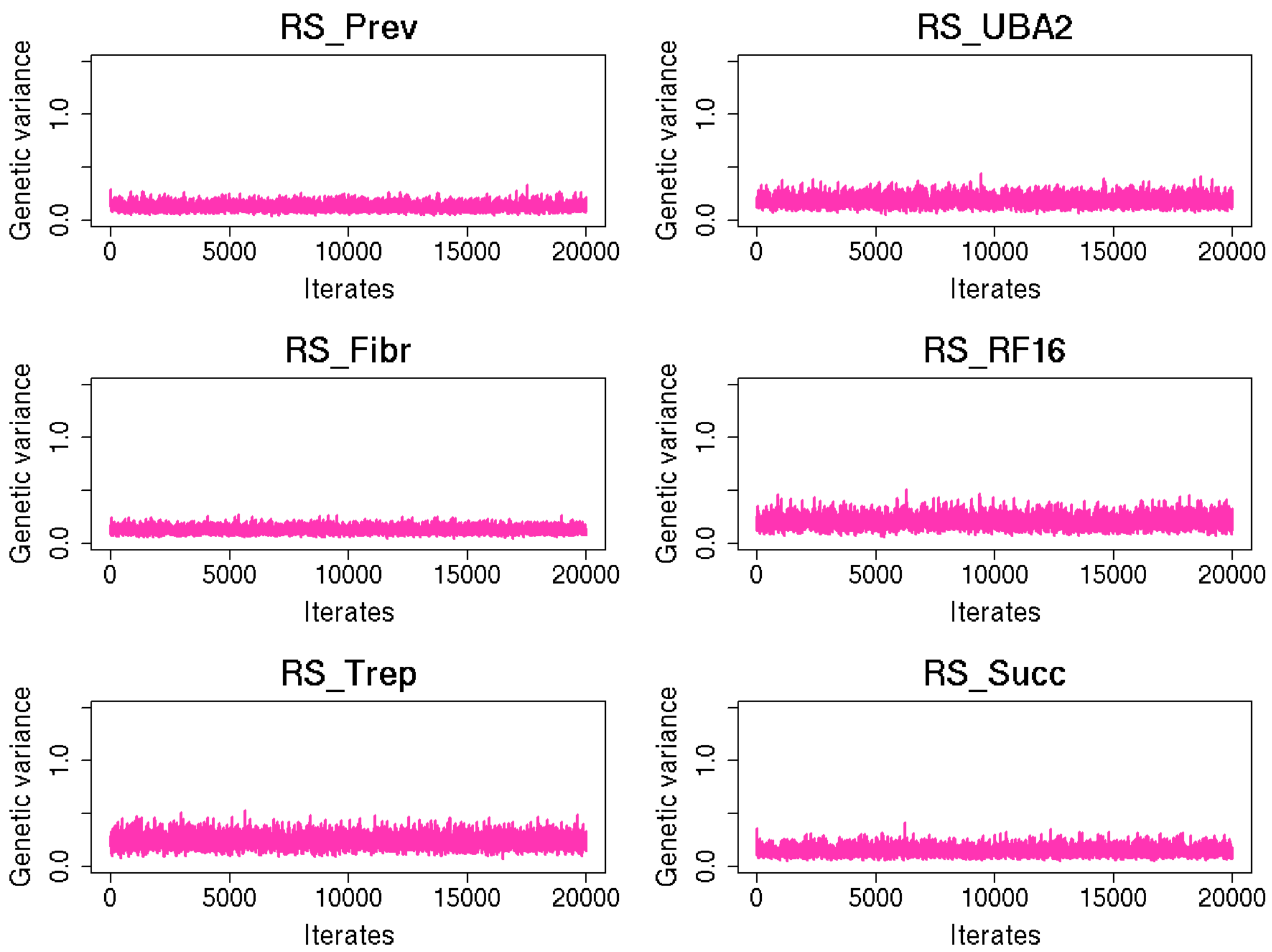


**Supplementary Figure 1:** Trace plots of genetic variances across iterations to show convergence for Holstein discovery set.

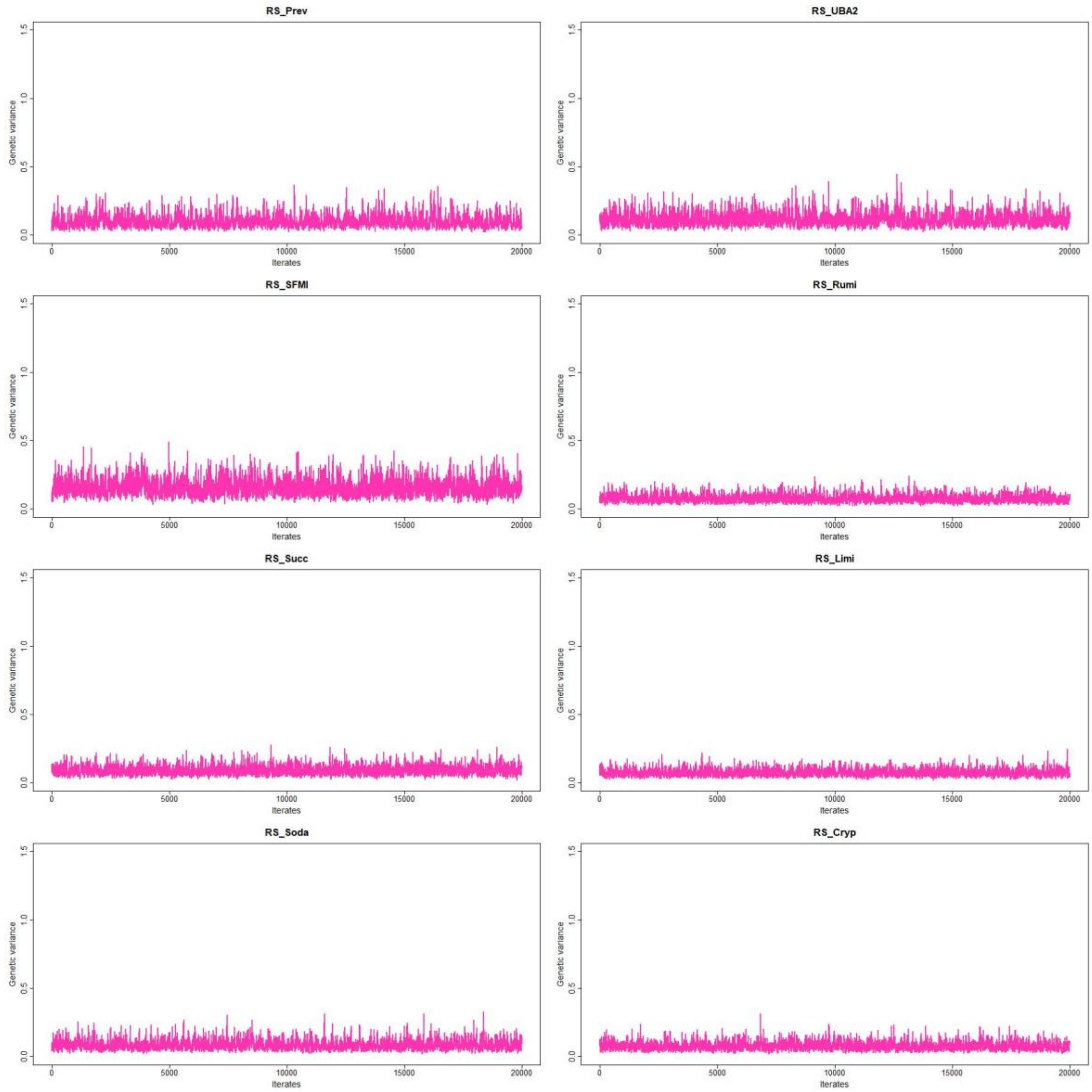


**Supplementary Figure 2:** Trace plots of genetic variances across iterations to show convergence for Canadian dataset.

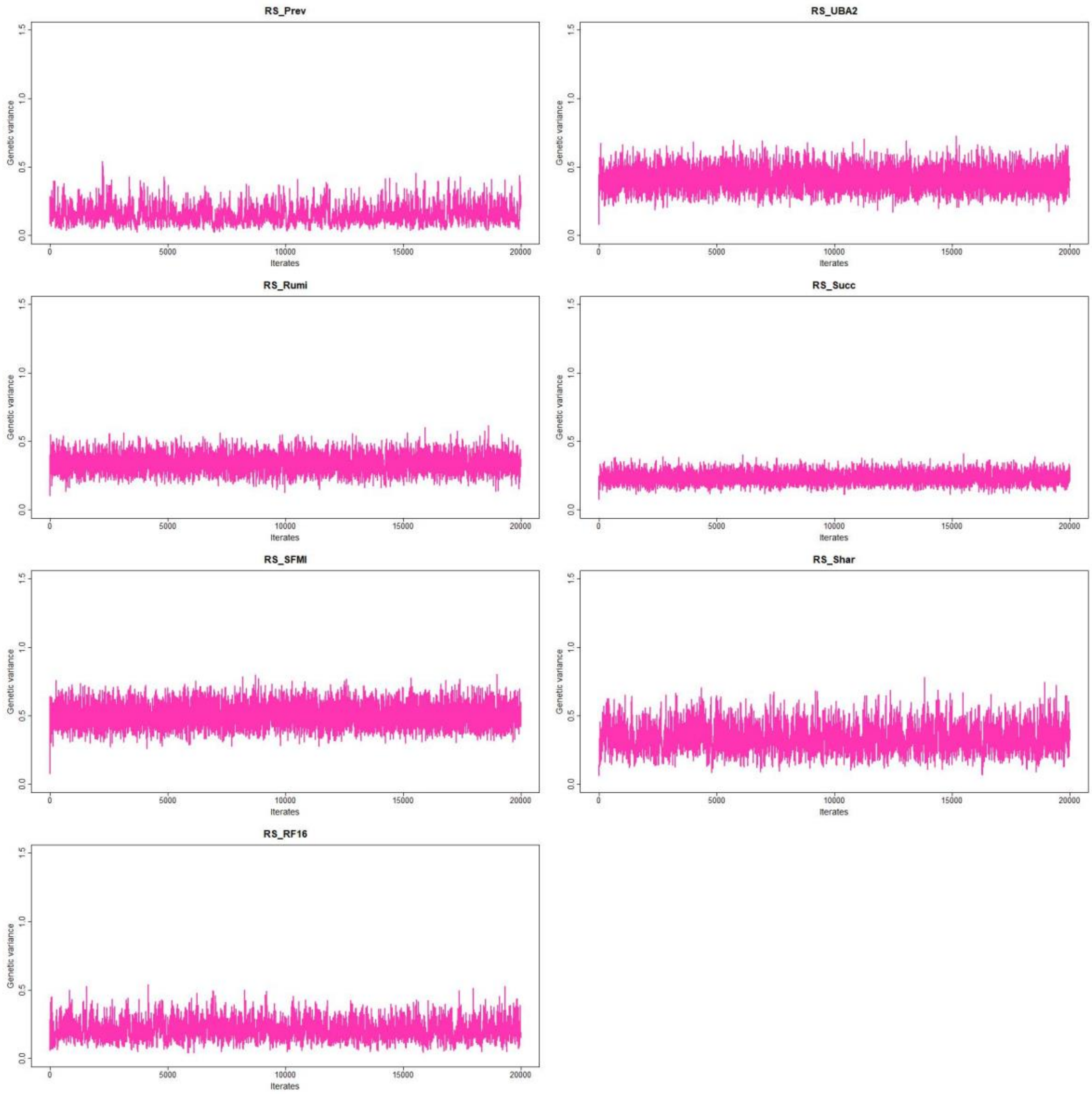


**Supplementary Figure 3** Trace plots of genetic variances across iterations to show convergence for Irish dataset.

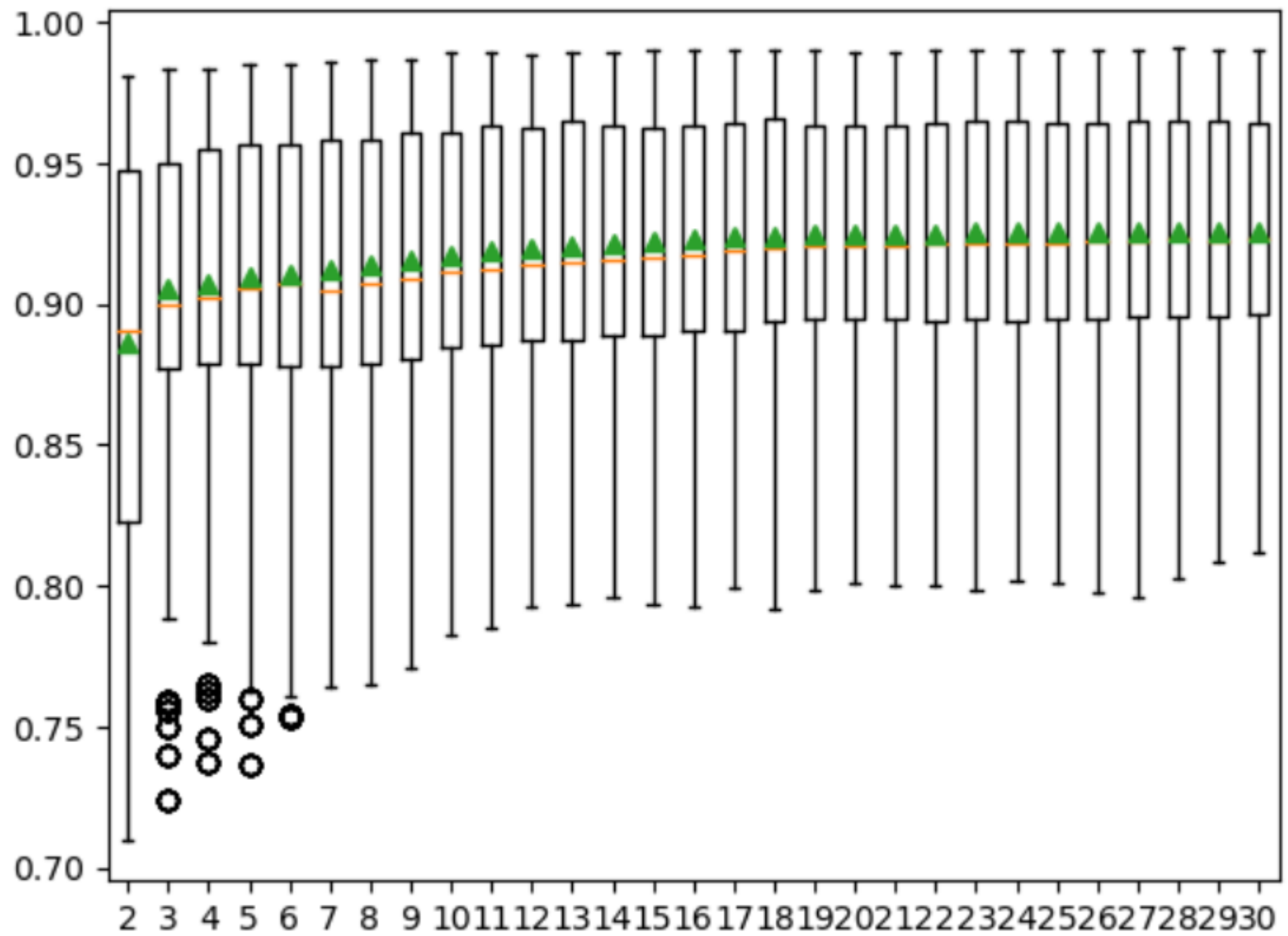


**Supplementary Figure 4:** Cosine similarity (y-axis) of the original genus abundances for Holstein discovery set, applying 3x3 bi-cross validation for a k range from 2 to 30 (x-axis).

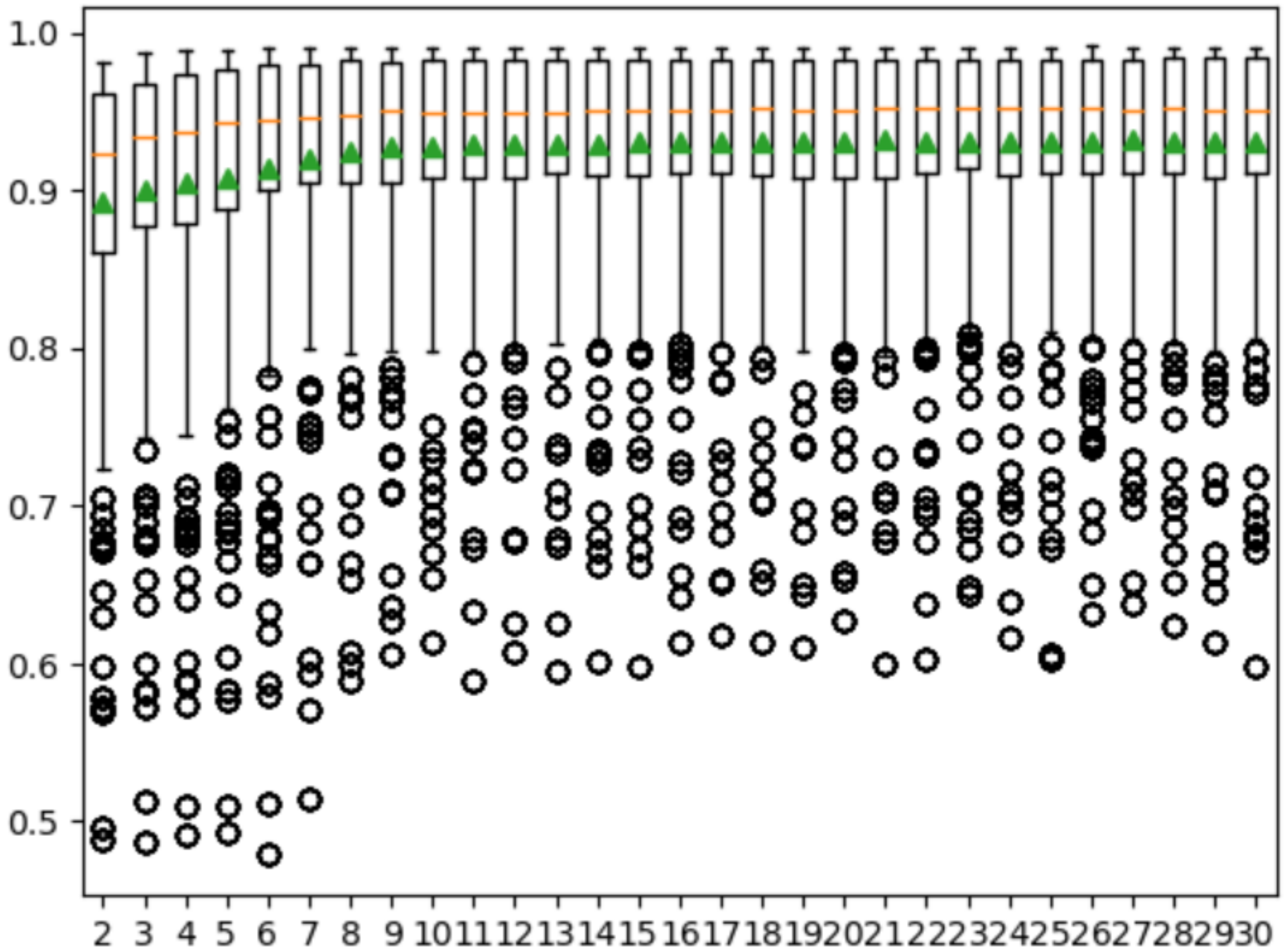


**Supplementary Figure 5:** Cosine similarity (y-axis) of the original genus abundances for Holstein validation set, applying 3x3 bi-cross validation for a k range from 2 to 30 (x-axis).

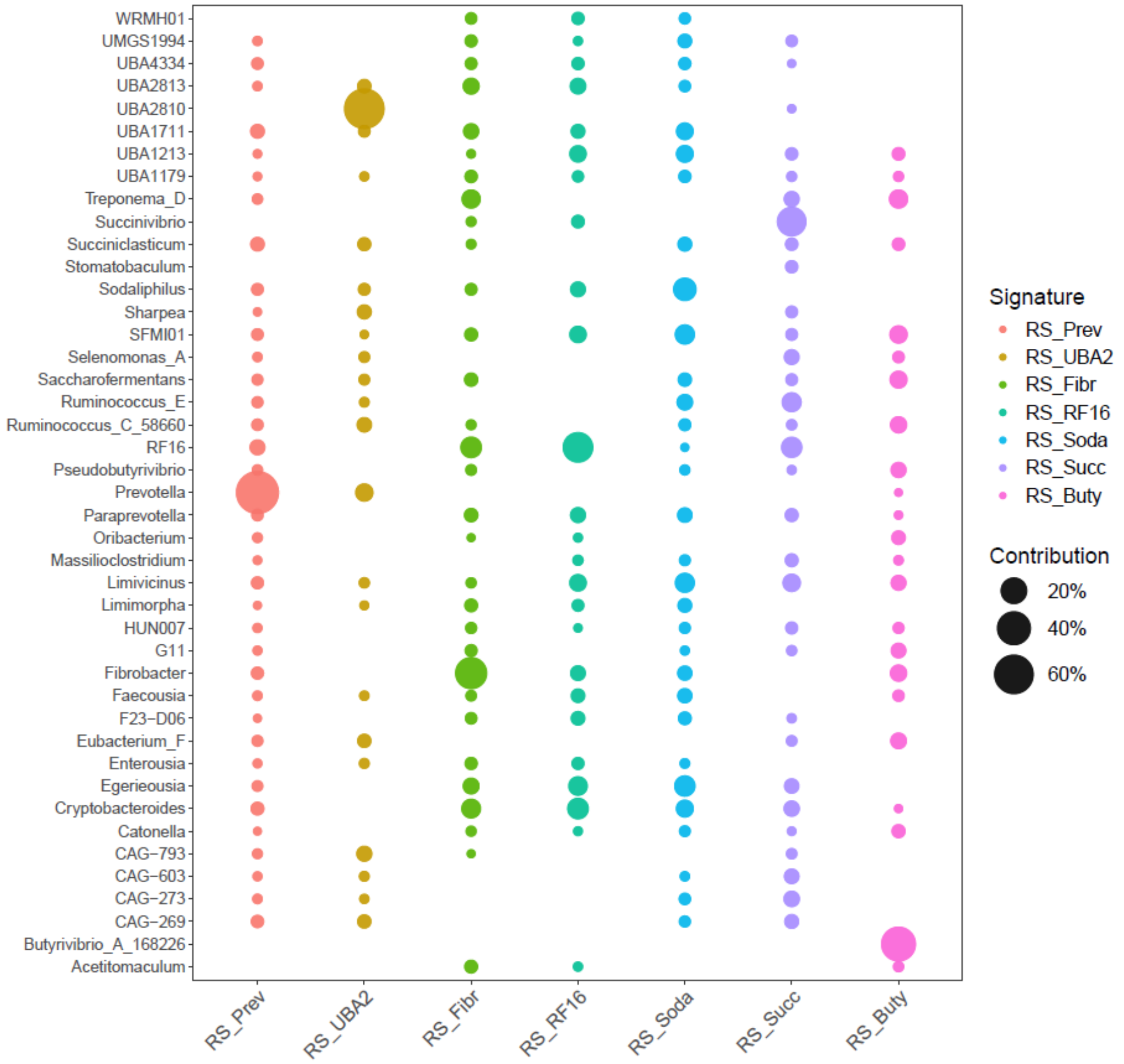


**Supplementary Figure 6:** Relative genera composition of RS in Holstein validation set. Only genera with a contribution higher than 1% in at least one of the RS are shown.

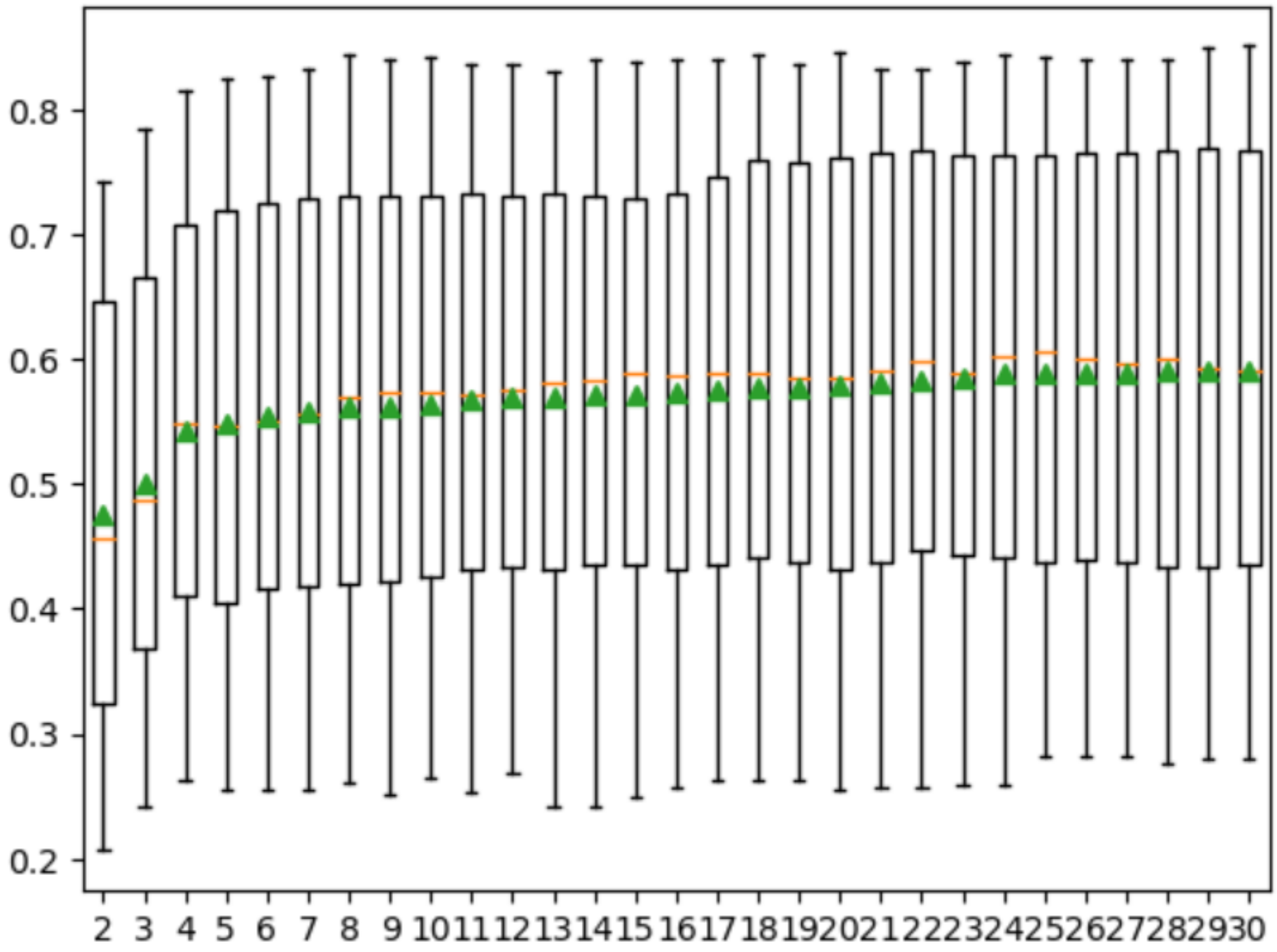


**Supplementary Figure 7:** Cosine similarity (y-axis) of the original genus abundances for Canadian dataset applying 3x3 bi-cross validation for a k range from 2 to 30 (x-axis).

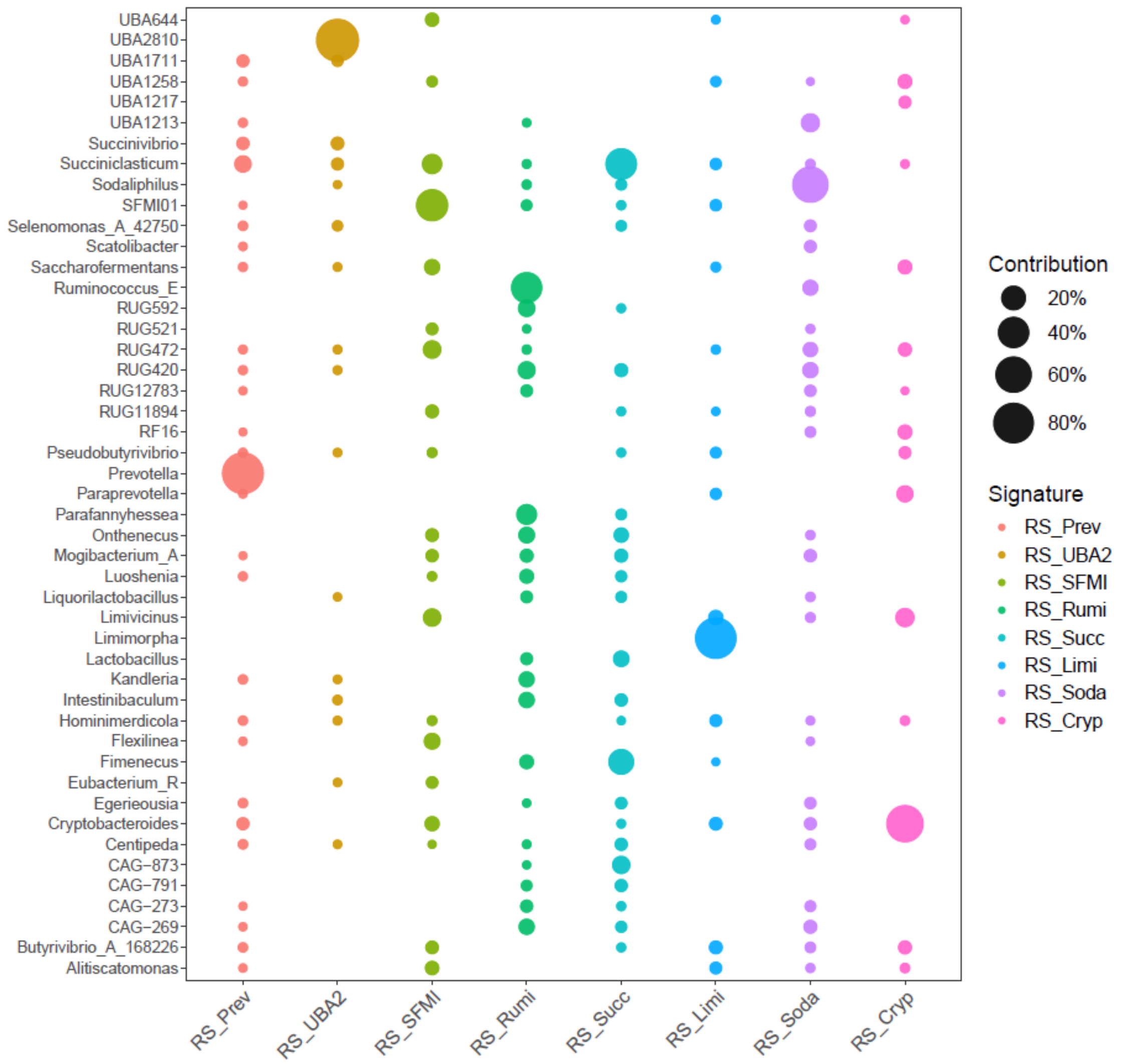


**Supplementary Figure 8**. Relative genera composition of Ruminosignatures in Canadian cattle. Only genera with a contribution higher than 1% in at least one of the Ruminosignatures are shown.

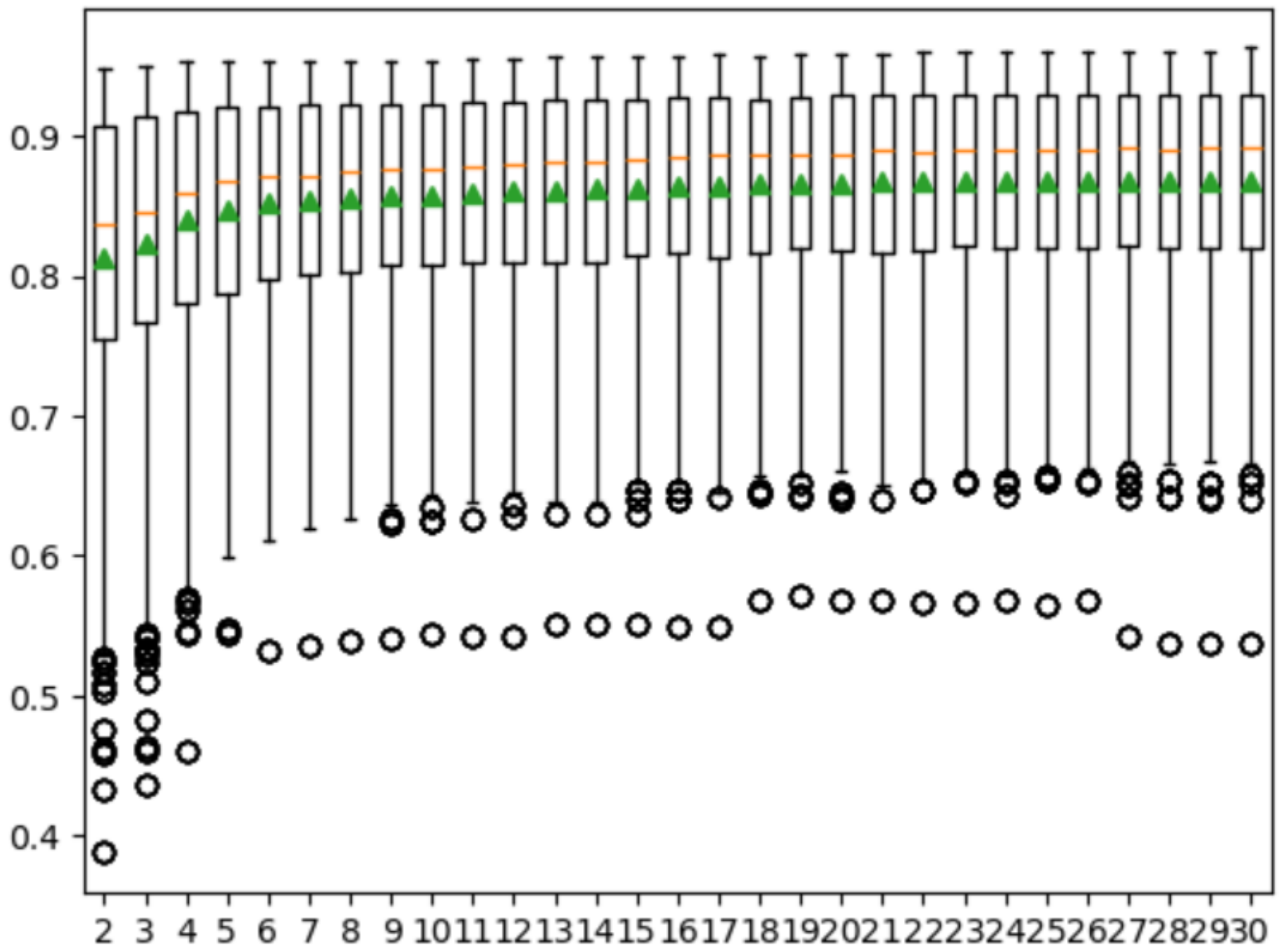


**Supplementary Figure 9:** Cosine similarity (y-axis) of the original genus abundances for Irish dataset applying 3x3 bi-cross validation for a k range from 2 to 30 (x-axis).

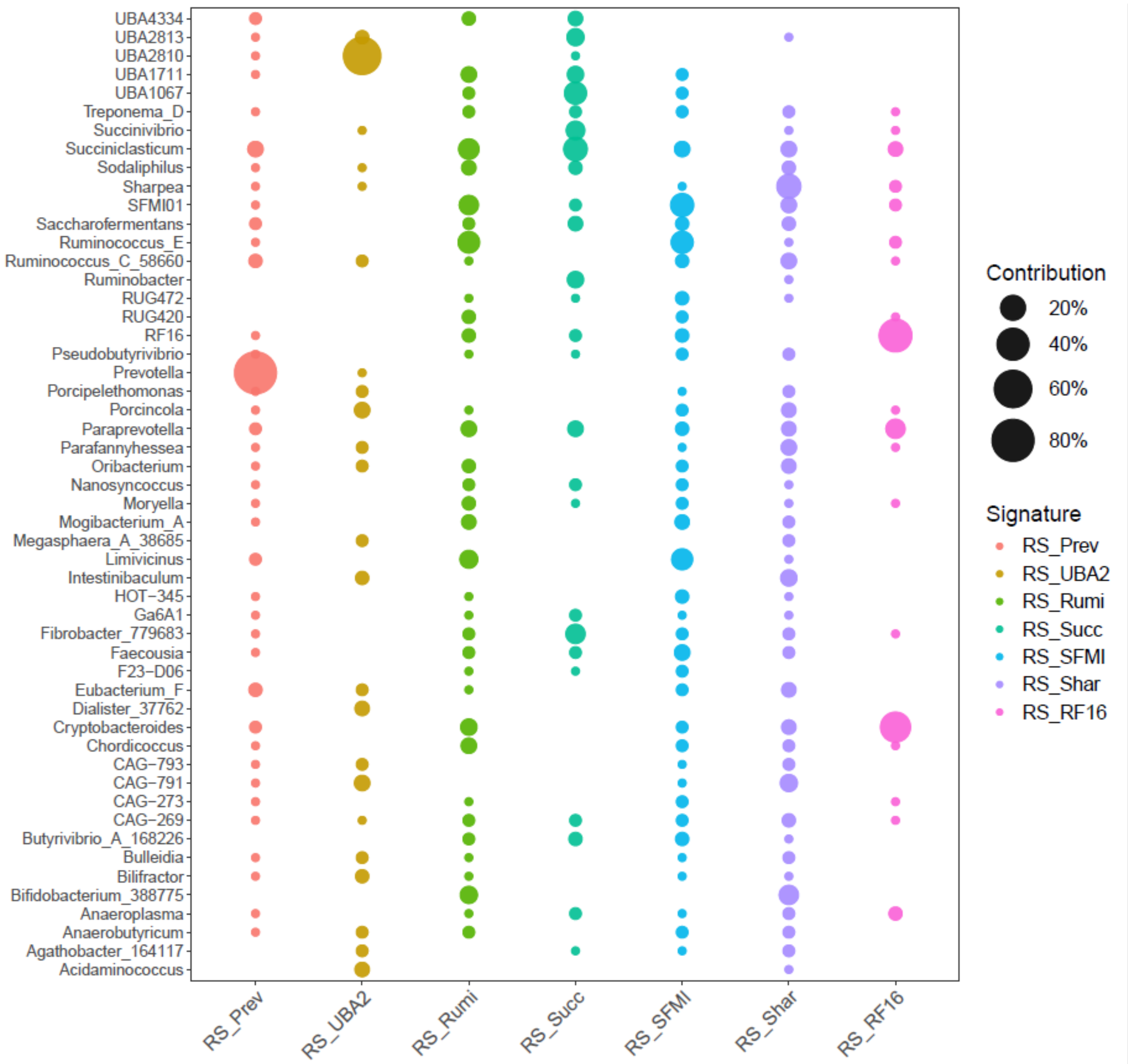


**Supplementary Figure 10.** Relative genera composition of Ruminosignatures in Ireland crossbred animals. Only genera with a contribution higher than 1% in at least one of the Ruminosignatures are shown.

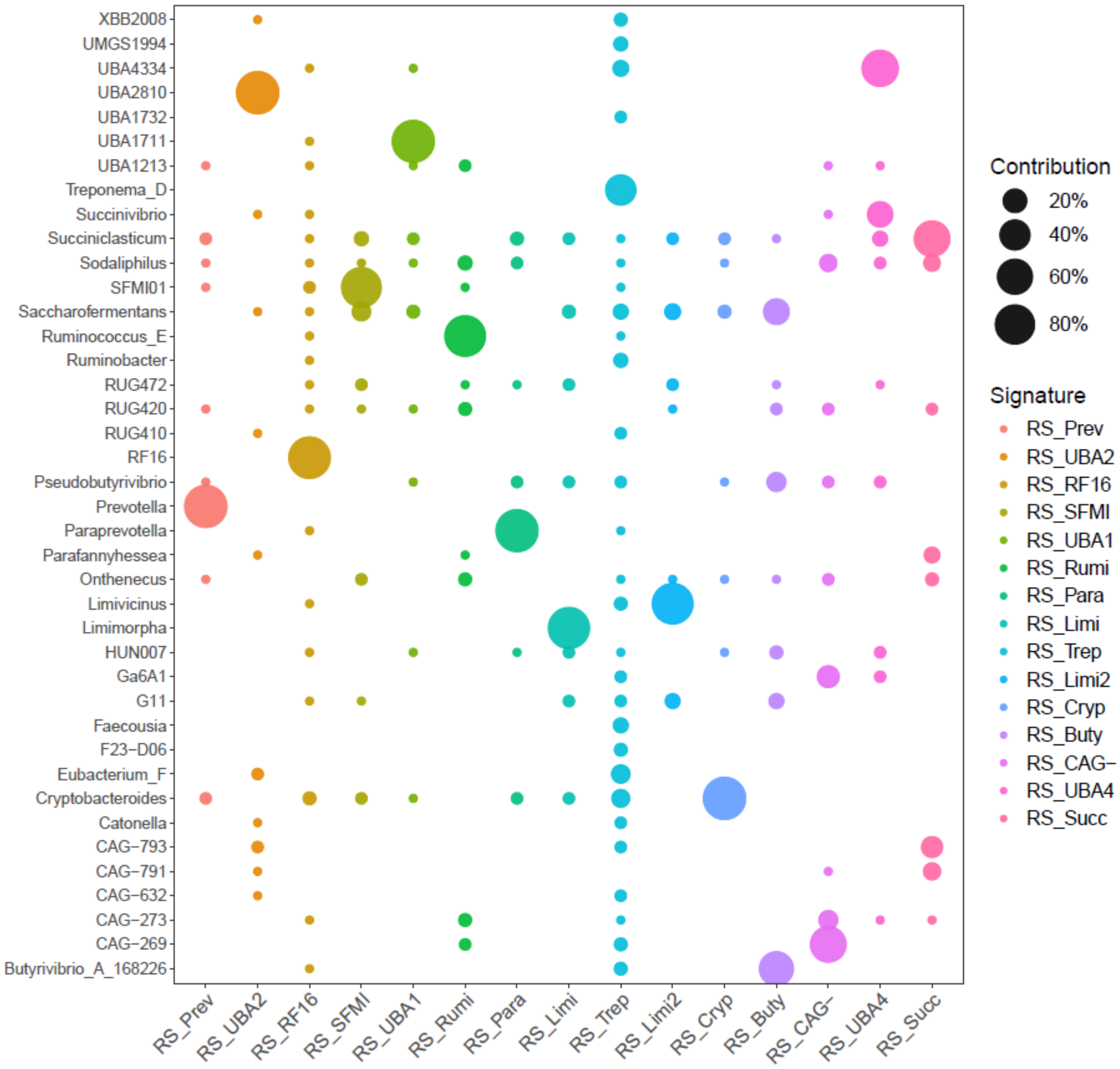


**Supplementary Figure 11:** Relative genera composition of Ruminosignatures in cross-cohort study. Only genera with a contribution higher than 1% in at least one of the Ruminosignatures are shown.

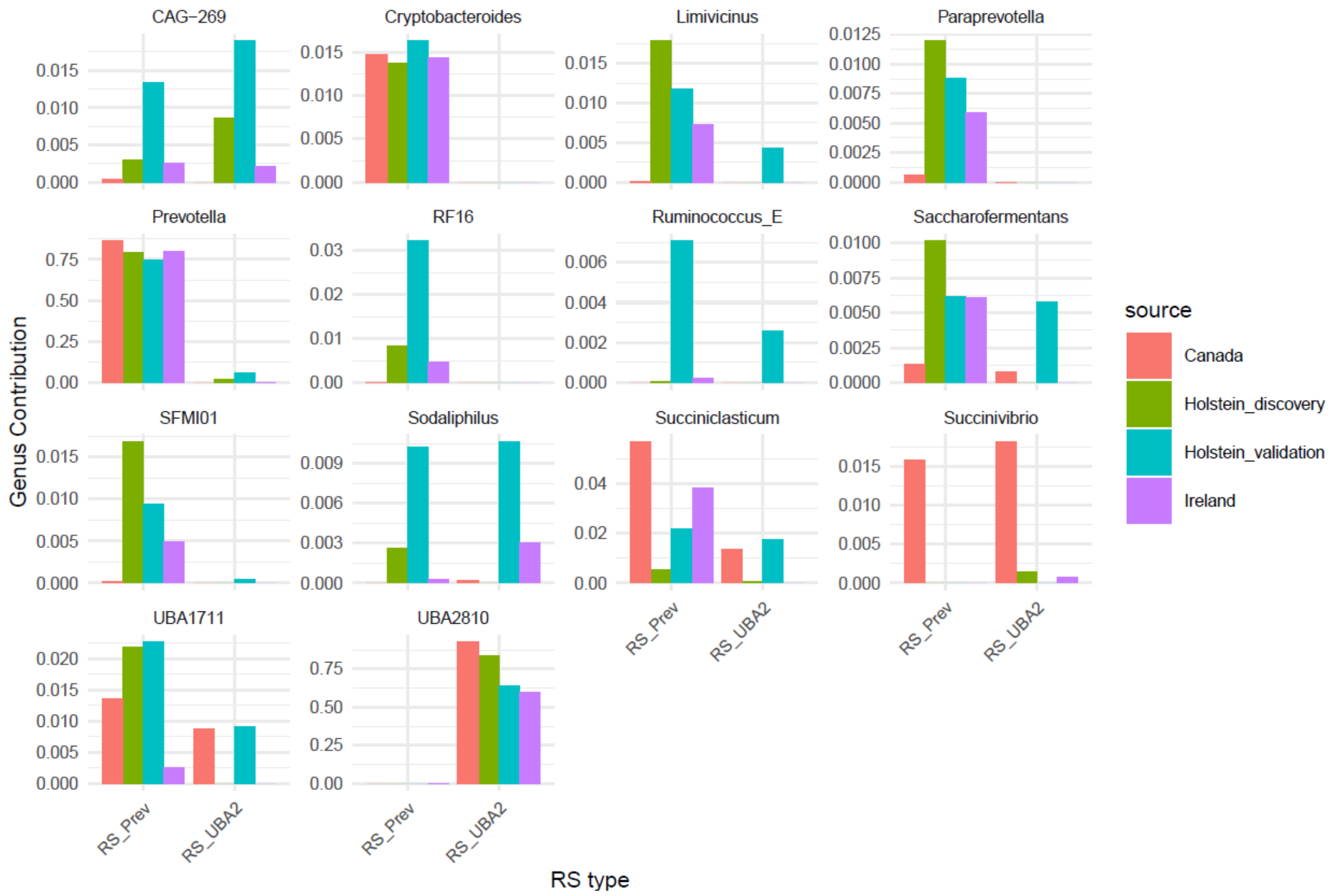


**Supplementary Figure 12:** The bar plots panel illustrates the contribution of fourteen common genera identified across the four datasets, with distributions shown separately for the two core microbia RSs, RS_Prev and RS_UBA2. The figure highlights the stability of dominant genera (e.g., Prevotella in RS_Prev, UBA2810 in RS_UBA2) alongside variation in the co-occurring taxa across datasets.

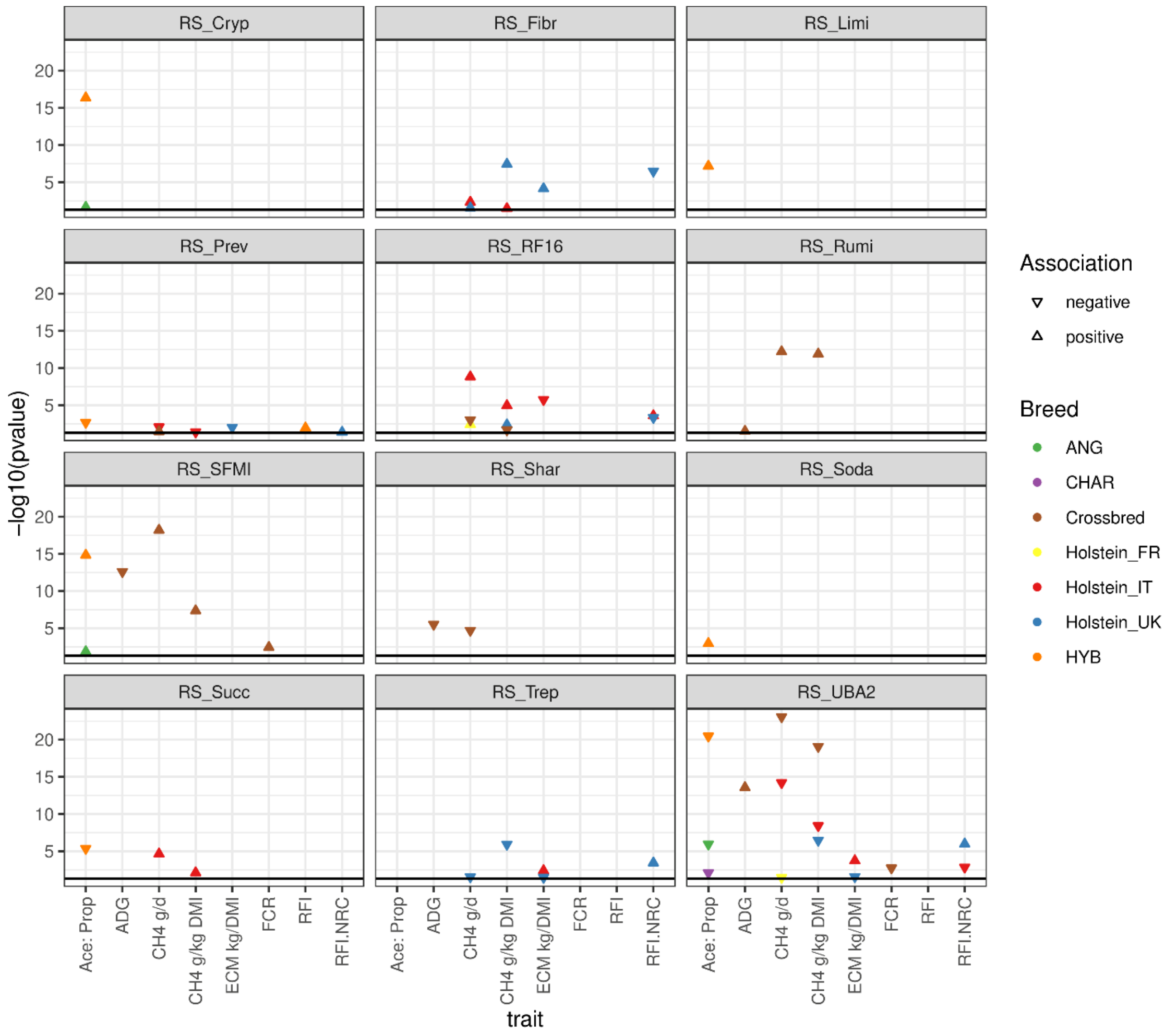


**Supplementary Figure 13:** Linear associations between Ruminosignatures abundances and methane or host performance traits. Arrows indicate the direction of the relationship between each Ruminosignatures and host trait. Only significant associations are shown, although the traits were measured in additional breeds not displayed in the plot.

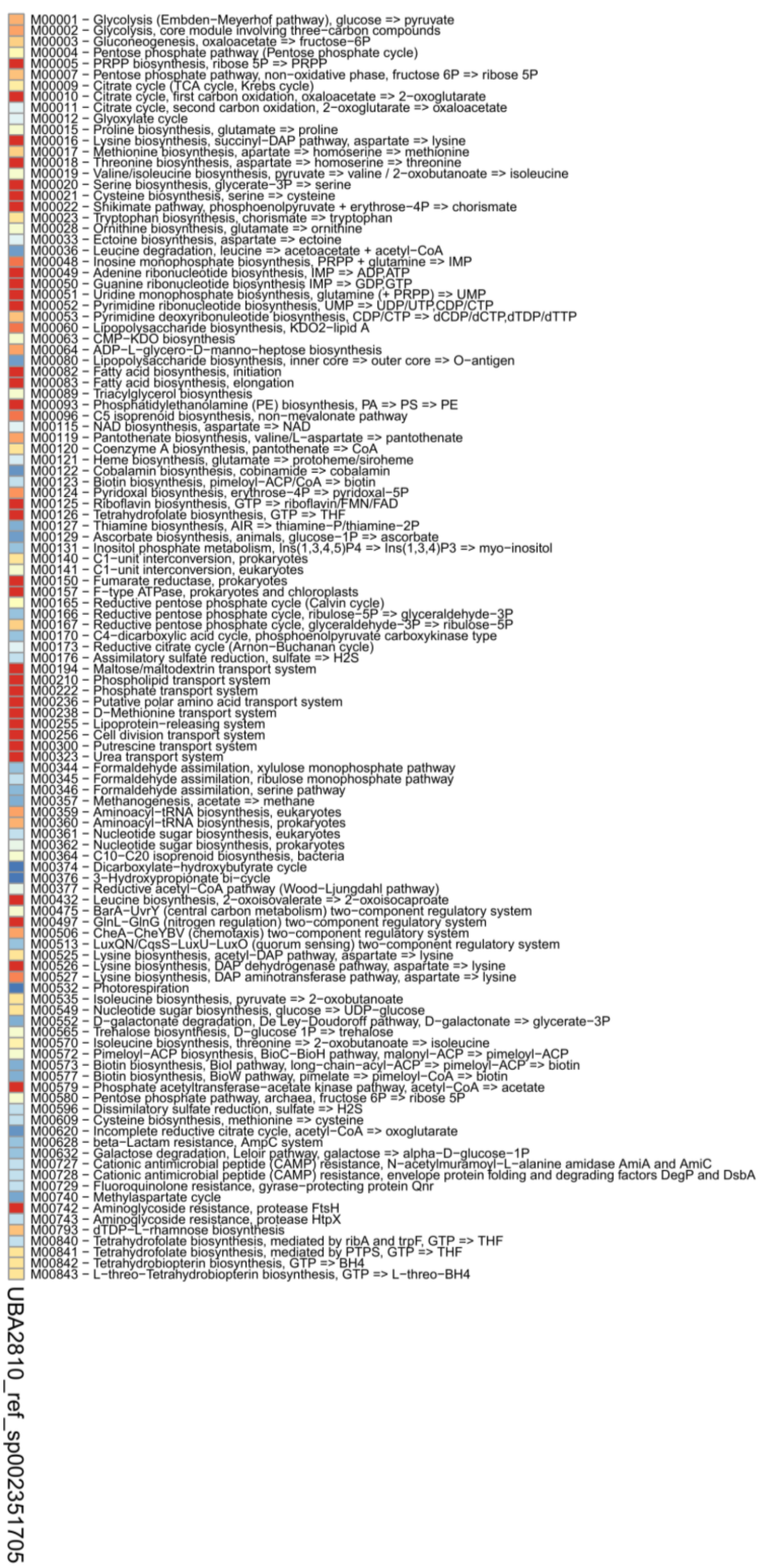

**Supplementary Figure 14:** KEGG module completeness predicted for UBA2810 sp002351705 using DRAM annotation and MetQy analysis. Colors indicate module completeness scores from low (blue) to high (red).

**REFERENCES**

1. R Core Team. R: a language and environment for statistical computing. Vienna: R foundation for statistical computing; 2025.
2. Wickham H. ggplot2: elegant graphics for data analysis. New York: Springer-Verlag; 2016.
3. Jombart T, Devillard S and Balloux, F. Discriminant analysis of principal components: a new method for the analysis of genetically structured populations. 2010. BMC Genetics 11: 94.
4. Jombart, T. adegenet: a R package for the multivariate analysis of genetic markers. Bioinformatics 2008. 24: 1403-1405
5. Sargolzaei M, Chesnais JP, Schenkel FS. A new approach for efficient genotype imputation using information from relatives. BMC Genomics. 2014;15(1):478.
6. Purcell, S., Neale, B., Todd-Brown, K., et al. PLINK: a tool set for whole-genome association and population-based linkage analyses. Am J Hum Genet. 2007;81, 559–575.
7. Gianola D, Fernando RL, Stella A. Genomic-assisted prediction of genetic value with semiparametric procedures. Genetics. 2006;173:1761–76.
8. Pérez P, de los Campos G. Genome-wide regression and prediction with the BGLR statistical package. Genetics. 2014;198:483–95.
9. VanRaden PM. Efficient methods to compute genomic predictions. J Dairy Sci. 2008;91:4414–23.
10. Amadeu RR, Cellon C, Olmstead JW, Garcia AA, Resende MF, Muñoz PR. AGHmatrix: R package to construct relationship matrices for autotetraploid and diploid species: a blueberry example. Plant Genome. 2016.
11. Plummer M, Best N, Cowles K, Vines K. CODA: convergence diagnosis and output analysis for MCMC. R News. 2006;6:7–11.
12. Oksanen J, Simpson GL, Blanchet FG, Kindt R, Legendre P, Minchin PR, O'Hara RB, Solymos P, Stevens MHH, Szoecs E, Wagner H, Barbour M, Bedward M, Bolker B, Borcard D, Carvalho G, Chirico M, De Caceres M, Durand S, Evangelista HBA, FitzJohn R, Friendly M, Furneaux B, Hannigan G, Hill MO, Lahti L, McGlinn D, Ouellette MH, Ribeiro Cunha E, Smith T, Stier A, Ter Braak CJF, Weedon J. 2024. vegan: Community Ecology Package.
13. Peraza P, Martinez-Boggio G, Naya H, Sotelo-Silveira J, Navajas EA. Predictive ability and mediation effects of the rumen microbiome on feed efficiency and methane traits in Hereford beef cattle. Animal. 2026 Jan;20(1):101730.
14. Sorensen, D., & Gianola, D. (2002). Likelihood of Bayesian, and MCMC Methods in Quantitative Genetics. In K. Dietz, M. Gail, K. Krickeberg, J. Samet, & A. Tsiatis (Eds.), *Crop science* ( 1st ed., Vol. **43**). New York, NY: Springer-Verlag, New York, Inc.
15. Aguilar, I, Tsuruta, S., Masuda, Y., Lourenco, D.A.L., Legarra, A., Misztal, I. 2018. BLUPF90 suite of programs for animal breeding. In: Proceedings of the 11th World Congress of Genetics Applied to Livestock Production, 11–16 February 2018, Auckland, New Zealand, p. 11.751.

16. Geweke J. Evaluating the accuracy of sampling-based approaches to the calculation of posterior moments. In: Bernardo JM, Berger JO, Dawid AP, Smith AFM (eds). Bayesian Statistics 4. Oxford University Press, Oxford. 1992;169–193.
17. Parks DH, Chuvochina M, Rinke C, Mussig AJ, Chaumeil PA, Hugenholtz P. GTDB: an ongoing census of bacterial and archaeal diversity through a phylogenetically consistent, rank normalized and complete genome-based taxonomy. Nucleic Acids Research 2021;50:D785–D794.
18. Shaffer M, Borton MA, McGivern BB, Zayed AA, La Rosa SL, Solden LM, Liu P, Narrowe AB, Rodríguez-Ramos J, Bolduc B, *et al*. DRAM for distilling microbial metabolism to automate the curation of microbiome function. *Nucleic Acids Research* 2020;48:8883–8900.
19. Konwar KM, Hanson NW, Hallam SJ. MetQy - an R package to query metabolic functions of genes and genomes. *Bioinformatics* 2016;32:3678–3679.
